# ETV1 activates a rapid conduction transcriptional program in rodent and human cardiomyocytes

**DOI:** 10.1038/s41598-018-28239-7

**Published:** 2018-07-02

**Authors:** Akshay Shekhar, Xianming Lin, Bin Lin, Fang-Yu Liu, Jie Zhang, Alireza Khodadadi-Jamayran, Aristotelis Tsirigos, Lei Bu, Glenn I. Fishman, David S. Park

**Affiliations:** 1 Leon H. Charney Division of Cardiology, New York University Langone Health, New York, New York, 10016 USA; 2 Center for Health Informatics and Bioinformatics, New York University Langone Health, New York, New York, 10016 USA

## Abstract

Rapid impulse propagation is a defining attribute of the pectinated atrial myocardium and His-Purkinje system (HPS) that safeguards against atrial and ventricular arrhythmias, conduction block, and myocardial dyssynchrony. The complex transcriptional circuitry that dictates rapid conduction remains incompletely understood. Here, we demonstrate that ETV1 (ER81)-dependent gene networks dictate the unique electrophysiological characteristics of atrial and His-Purkinje myocytes. Cardiomyocyte-specific deletion of ETV1 results in cardiac conduction abnormalities, decreased expression of rapid conduction genes (*Nkx2–5*, *Gja5*, and *Scn5a*), HPS hypoplasia, and ventricularization of the unique sodium channel properties that define Purkinje and atrial myocytes in the adult heart. Forced expression of ETV1 in postnatal ventricular myocytes (VMs) reveals that ETV1 promotes a HPS gene signature while diminishing ventricular and nodal gene networks. Remarkably, ETV1 induction in human induced pluripotent stem cell-derived cardiomyocytes increases rapid conduction gene expression and inward sodium currents, converting them towards a HPS phenotype. Our data identify a cardiomyocyte-autonomous, ETV1-dependent pathway that is responsible for specification of rapid conduction zones in the heart and demonstrate that ETV1 is sufficient to promote a HPS transcriptional and functional program upon VMs.

## Introduction

Rapidly conducting atrial and Purkinje cells (PC) and slowly conducting, impulse generating nodal cells cooperate to coordinate the heartbeat^1^. As a result, vertebrate animals establish a primitive conduction hierarchy during embryonic development concurrently with the formation of the early heart tube using highly conserved genetic pathways^2–6^. Rapid conduction myocytes form in pectinated and trabecular regions during cardiac chamber formation and mature into the pectinated atrial myocardium (PAM) and the His-Purkinje system (HPS), respectively. Rapid cardiac conduction is essential for normal rhythm maintenance^7,8^ and optimization of synchronous contraction of the cardiac chambers. Consequently, dysregulation of rapid conduction regions produce a large burden of arrhythmic diseases, including atrial fibrillation and Purkinje-mediated ventricular arrhythmias^7–10^. In addition, conduction block in the HPS is associated with increased mortality in heart failure patients due to myocardial dyssynchrony. Despite their clinical importance, gene regulatory networks (GRNs) that establish rapid conduction in the heart remains poorly defined. In particular, PCs remain the least characterized of all cardiac cell types due in part to their low abundance in the adult heart, comprising less than 1% of the total cell population^11^.

Seminal work revealed that the His-Purkinje system (HPS) originates from a cardiomyocyte lineage of trabecular myocytes during heart development^12–15^. Trabecular formation occurs though endocardial-derived neuregulin-1 (NRG1) signaling to cardiomyocytes through its cognate receptor complex ErbB4 and ErbB2^16^. Targeted deletion of *Nrg1*, *Erbb4*, or *Erbb2* results in the absence of the rapidly conducting trabecular zone in the mouse heart^16–18^. Moreover, NRG1 is sufficient to convert developing cardiomyocytes towards a rapid conduction cell fate^19–21^. These studies reinforce that NRG1 dependent signaling engage gene regulatory networks (GRNs) within cardiomyocytes, which are responsible for trabecular and subsequent HPS development.

A mounting body of evidence now shows that cell type-enriched transcription factors (TFs) orchestrate rapid impulse conduction GRNs in these specialized cardiomyocytes. A handful of TFs important for PC specification and function have been identified, including *Nkx2-5*^22–25^, *Tbx5*^26,27^, *Tbx3*^28–30^, *Id2*^31^, *Hopx*^32^, *Irx3*^33,34^, *Irx5*^35,36^, and *Etv1*^21^, however their transcriptional abundance, cardiomyocyte-autonomous molecular function, and requirement for adult cardiac conduction health remain incompletely investigated. What has been clearly demonstrated is these TFs influence PCs to acquire a unique gene signature, compared to surrounding ventricular myocytes (VMs), through the expression of specialized ion channels and gap junction proteins^37^. Specifically, the enriched expression of the voltage-gated sodium channel protein Na_V_1.5 (encoded by *Scn5a*) and the gap junction channel protein Cx40 (encoded by *Gja5*) enable rapid conduction within the developing and mature heart^38–40^. Recently, we identified a critical role for the TF ETV1 in NRG1-dependent regulation of fast conduction biology^21^. ETV1, a member of the Pea3 group of ETS family TFs, is a transcriptional activator regulated through Ras-MAPK-dependent pathways in multiple cells types and functions at the cross roads between cellular proliferation, differentiation, and patterning^41–46^. In the heart, ETV1 is highly expressed in the developing and mature HPS and pectinated atrial myocardium (PAM)^21^. Previously, we showed in a germline knockout model that ETV1 is necessary for the expression of fast conduction genes (*Nkx2–5*, *Gja5*, *Scn5a*), determines the unique biophysical properties of the sodium current in atrial and PCs, and promotes proper formation of the HPS in the adolescent mouse heart^21^. In addition, we found that an *ETV1* sequence variant associates with bundle branch and atrioventricular blocks in humans^21^. Taken together, these data identified ETV1 as a critical regulator of the HPS gene program in cardiomyocytes. However, several questions remain unanswered, including whether the effects of ETV1 knockout reflect a cell-autonomous defect in myocytes, and whether ETV1 is sufficient to reprogram a fast conduction cell fate on rodent and human cardiomyocytes. In this study, we investigated the cardiomyocyte-specific role of ETV1 in regulation of PC specification, rapid conduction GRNs, and electrophysiology in murine and human model systems.

## Results

### Etv1 defines progressive maturation of rapid conduction zones in the heart

**Figure 1:**
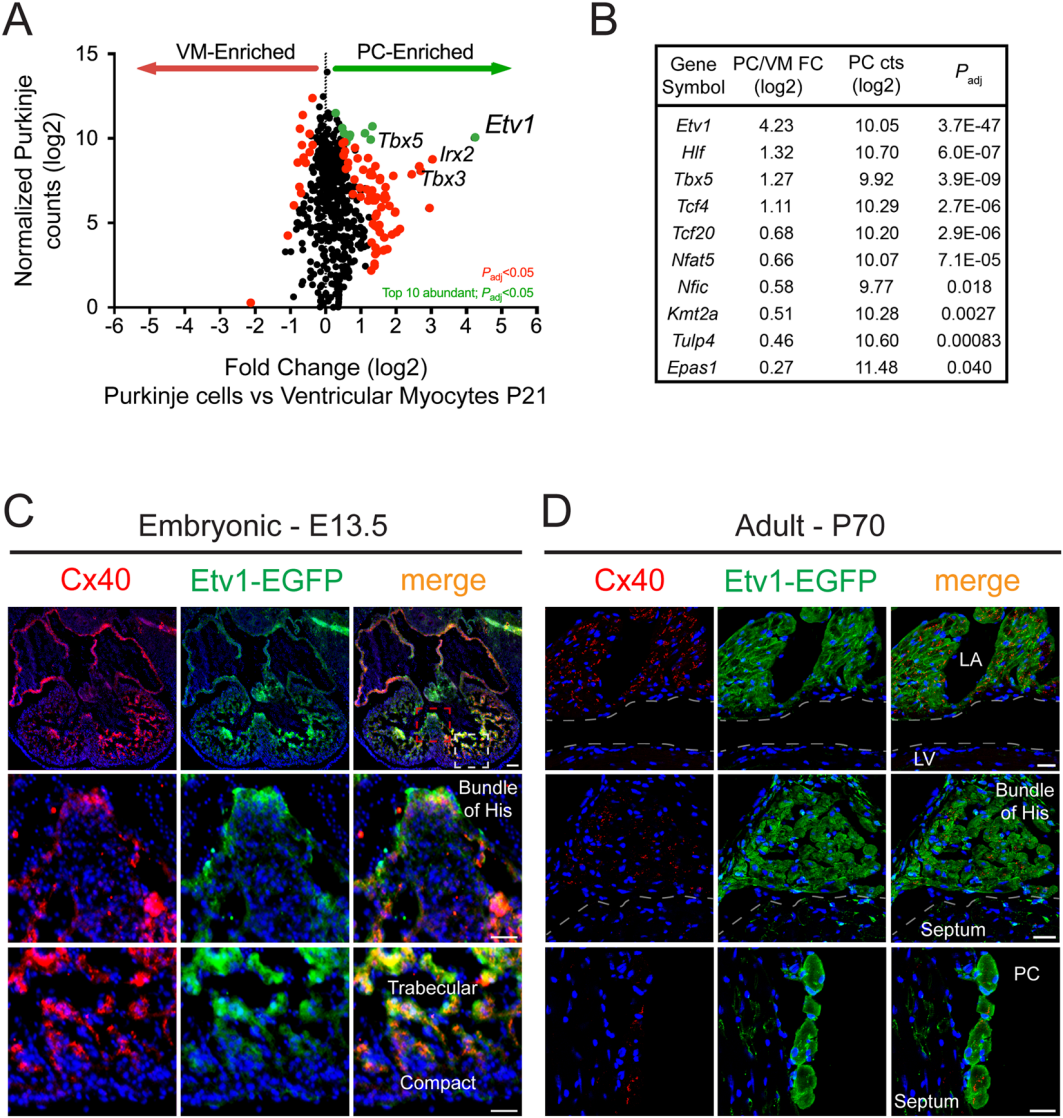
RNA Sequencing of fluorescence-activated cell sorted (FACS) postnatal day 21 (P21) mouse Purkinje cells (PC) and ventricular myocytes (VM). (**A**) Expression of PC (n = 7) and VM (n = 10) transcription factors (TFs) at P21. Differential expression (PC vs VM) on the X-axis and Purkinje transcript levels (normalized counts) are shown on the Y-axis. Significantly enriched (P_adj_ < 0.05) and top ten abundant PC-TFs labeled in green while all remaining significantly different TFs were labeled in red. TFs that were not significantly different labeled in black. (**B**) Top ten significantly enriched and abundant TFs in PCs. *Etv1* was the most enriched and one of the most abundant TFs within PCs. (**C**,**D**) *Etv1*-enhanced green fluorescent protein (EGFP) bacterial artificial chromosome (BAC) transgenic mice were used to confirm regional expression of *Etv1* in embryonic (**C**) and adult (**D**) hearts. (**C**) Immunohistochemistry on E13.5 *Etv1*-EGFP hearts showing overlapping expression of Connexin-40 (Cx40) and GFP in trabecular and atrial myocytes. Bottom two rows are high-magnification views of the Bundle of His (red dashed box) and Trabecular/Compact zone myocytes (white dashed box) outlined in the top row (**D**) Immunohistochemistry on P70 *Etv1*-EGFP hearts showing overlapping expression of Cx40 and GFP in atrial myocytes (top row), bundle of His (middle row), and PCs (bottom row). Scale bars: 100 μm (top) 50 μm (middle, bottom) (**C**); 25 μm (**D**).

Specific genetic markers of the HPS have facilitated investigation of the molecular circuitry regulating PC specification, patterning, and function^48–54^. Making use of a *Cntn2*-EGFP BAC genetic reporter^53^, we developed and verified a fluorescence-activated cell sorting (FACS)-based purification strategy to isolate PCs and surrounding VMs (Supplemental Fig. 1)^47^. Leveraging advances in next generation sequencing technology, we performed genome-wide RNA-seq on dissociated ventricles from postnatal day 21 (P21) wild type (WT) mice in a *Cntn2*-EGFP transgenic background. RNA-seq differential expression analysis between PCs and VMs identified 2,161 differentially expressed protein-coding genes from a total of 12,496 genes with detectible counts in cardiomyocytes (normalized counts > 5). A Kyoto Encyclopedia of Genes and Genomes (KEGG) functional analysis on the 1,336 PC enriched genes revealed an overrepresented number of neuronal gene networks, suggesting TFs and effectors that regulate neuronal development may play a role in cardiac PC development (Supplemental Fig. 2). Of all TFs, *Etv1* was the most differently expressed transcript in PCs compared to VMs (Fig. 1A). *Tbx3*, *Tbx5*, and *Irx2* were also differentially expressed between PCs and VMs in line with previous reports (Fig. 1A)^26,30,55^. *Etv1* was among the most abundant, significantly enriched PC TFs, along with *Tbx5* (Fig. 1B).

To verify cell-type specific expression of *Etv1* in the heart, we utilized *Etv1*-EGFP BAC transgenic reporter mice^56^. Examination of embryonic day 13.5 (E13.5) and P70 *Etv1*-EGFP sections showed GFP expression in rapid conduction regions of the developing and adult heart in agreement with other *Etv1*-reporter mice (Fig. 1C,D). At E13.5, *Etv1* was expressed specifically in Cx40-positive atrial myocytes (AM), bundle of His, and trabecular myocytes (Fig. 1C). Interestingly, we observed a transmural gradient of *Etv1*-EGFP expression in the developing ventricles with the highest expression at the trabeculae tips adjacent to the endocardium, the region of terminal PC specification (Fig. 1C, lower panels). In the adult heart, *Etv1* expression remained in Cx40-positive AM and the bundle of His (Fig. 1D). Ventricular *Etv1* expression was restricted to Cx40-positive mature PCs and absent in VMs (Fig. 1D, lower panels).

### Cardiomyocyte-specific ETV1 deletion causes cardiac conduction defects

**Figure 2:**
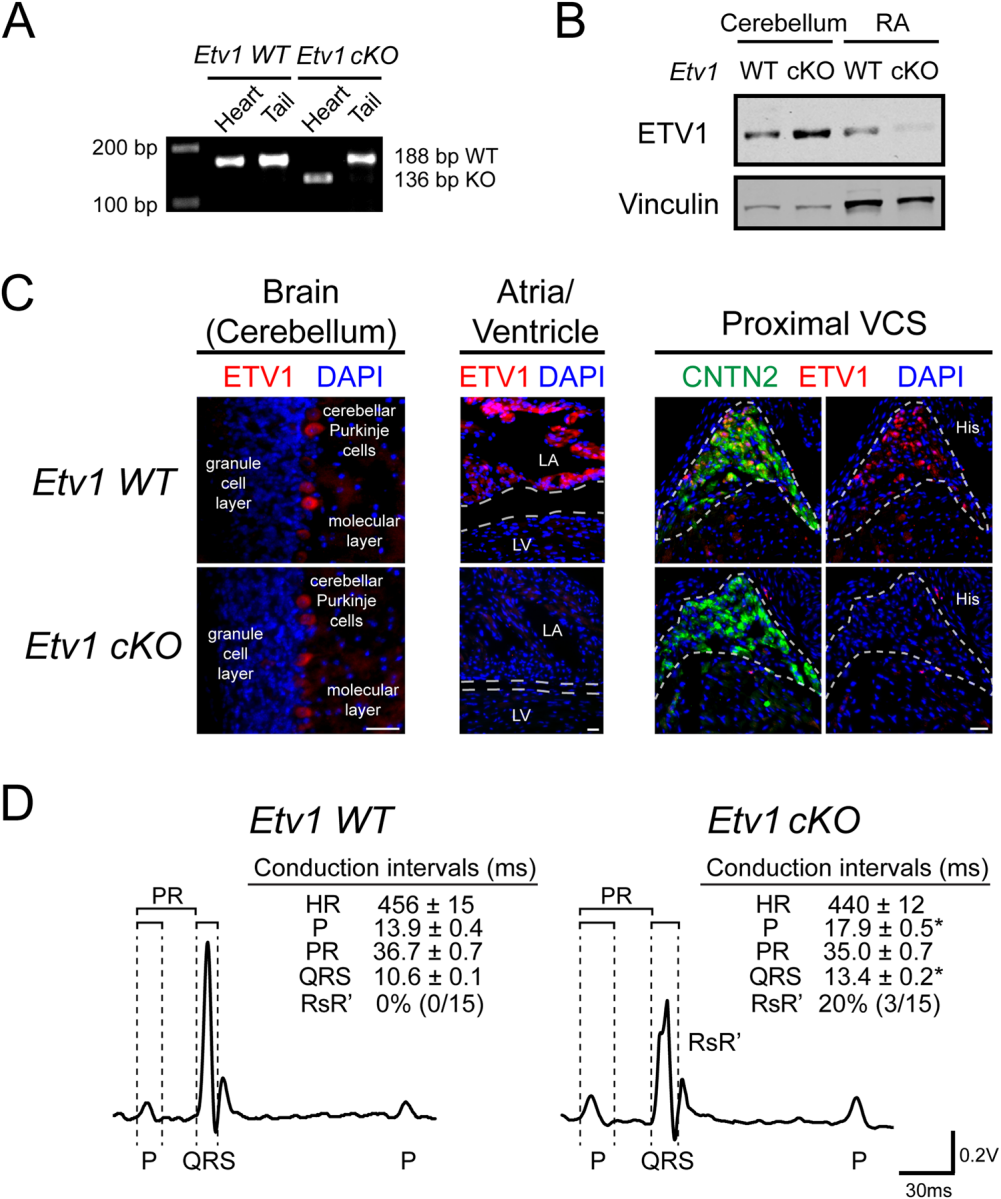
Cardiomyocyte-specific knockout of ETV1 slows atrial and His-Purkinje system conduction. *Etv1*^*flox/flox*^ mice crossed with *Myh6-Cre* transgenic mice enabled generation of cardiac-specific *Etv1* mutant mice. (**A**) Heart and tail RT-PCR analysis of DNA from *Etv1* WT (*Etv1*^*flox/flox*^) and *Etv1* cKO (*Etv1*^*flox/flox*^, *Myh6-Cre*) mice demonstrate heart specific deletion of *Etv1* exon 11. (**B**) Immunoblot assessment of *Etv1* WT and *Etv1* cKO tissue extracts. Protein from cerebellum and right atrial lysates were electrophoresed and immunoblotted to detect ETV1 (top) and Vinculin (bottom). (**C**) Immunofluorescence evaluation of ETV1 expression in 10-week-old *Etv1* WT and *Etv1* cKO cerebellar Purkinje cells, atrial myocytes, and His-Purkinje system (HPS). Positive CNTN2 expression identified HPS cells. Nuclei were identified by DAPI (blue). (**D**) Representative surface ECG traces of 10-week-old *Etv1* WT and *Etv1 c*KO mice. Conduction intervals from 10–12-week-old *Etv1* cKO mice compared to *Etv1* WT mice showed significantly prolonged P wave and QRS intervals. 20% of *Etv1* cKO mice displayed an RsR’ pattern. LA, left atria; LV, left ventricle. Data represent mean ± SEM. Scale bars: 25 um.

Germline *Etv1*-deficient mice are runted and die by three-weeks of age due to a progressive neuromuscular defect, limiting cardiac electrophysiological assessments at adult stages^43^. To circumvent these shortcomings, we generated cardiomyocyte-specific *Etv1* null mice by crossing *Etv1-*floxed mice^57^ with transgenic *Myh6-Cre* mice, which express Cre recombinase under the regulatory sequences of the cardiac alpha (α)-myosin heavy chain gene^58^, hereby referred to as *Etv1* cKO mice (*Etv1*^*fl/fl*^; *Myh6*-*Cre*). Genomic DNA analysis revealed that *Etv1* exon 11 was specifically deleted in the heart of *Etv1* cKO mice, but unaltered in non-cardiomyocytes (Fig. 2A). Absence of ETV1 protein was confirmed via immunoblots demonstrating specific loss of right atrial ETV1 expression in *Etv1 cKO* mouse hearts while ETV1 expression remained preserved in cerebellar brain and WT (*Etv1*^fl/fl^) tissues (Fig. 2B). Immunofluorescence of *Etv1* WT and cKO heart and brain sections confirmed cell-type specific absence of ETV1 in AM and HPS while expression was unaltered in cerebellar Purkinje cells (Fig. 2C).

In order to examine the effect of *Etv1* deletion in the adult heart, echocardiograms and electrocardiograms (ECGs) were performed on anesthetized 10–12-week-old *Etv1* WT and cKO mice. *Etv1* cKO mice survive to adulthood and maintain normal body weight in contrast to *Etv1* germline KO mice (Data not shown). Cardiomyocyte loss of ETV1 resulted in no significant differences in cardiac size or contractility (Supplemental Table 1), however surface ECG of *Etv1* cKO mice revealed significant electrophysiological deficits. *Etv1 c*KO mice had prolonged P wave duration and a widened QRS interval with a 20% prevalence of bundle branch block (RsR’) (Fig. 2D, Supplemental Fig. 3, Supplemental Table 2). RsR’ pattern was not observed in *Etv1* WT animals.

### Cardiomyocyte-specific ETV1 deletion disrupts expression of genes required for rapid conduction

**Figure 3:**
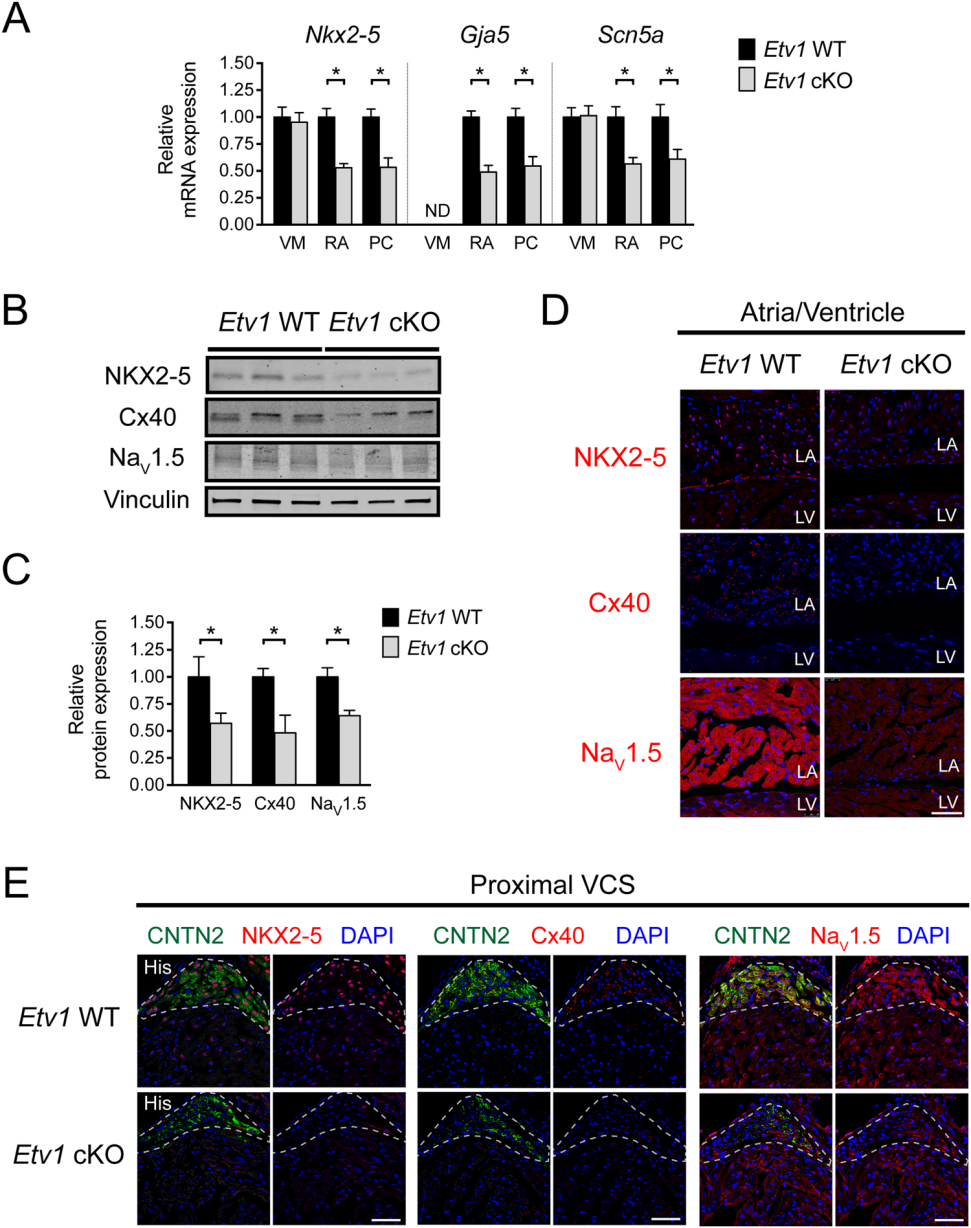
Cardiomyocyte deletion of ETV1 resulted in decreased expression of fast conduction genes in atrial and His-Purkinje system (HPS) myocytes. (**A**) Quantitative RT-PCR of fast conduction gene RNA levels (normalized to *Gapdh*) comparing 10–12-week-old *Etv1* WT (*Etv1*^*flox/flox*^) and *Etv1* cKO (*Etv1*^*flox/flox*^, *Myh6-Cre*) FACS-purified ventricular, atrial, and Purkinje myocytes. Relative *Nkx2–5*, *Gja5*, and *Scn5a* expression displayed versus control, *Etv1* WT (n = 4). (**B**) Immunoblot assessment of *Etv1* WT and *Etv1* cKO atrial tissue lysates detecting NKX2–5, Cx40, Na_V_1.5, and Vinculin (loading control). (**C**) Protein level densitometric quantification (normalized to vinculin), displayed relative to *Etv1* WT (n = 5). (**D**) Immunofluorescence evaluation of NKX2–5, Cx40, and Na_V_1.5 expression in 10-week-old *Etv1* WT and *Etv1* cKO atria/ventricular sections. (**E**) Immunofluorescence evaluation of NKX2–5, Cx40, and Na_V_1.5 expression in 10-week-old *Etv1* WT and *Etv1* cKO HPS sections. Positive CNTN2 expression identified HPS cells. Nuclei were identified by DAPI (blue). LA, left atria; LV, left ventricle. Data represent mean ± SEM. *P < 0.05, 2-tailed Student’s t test. Scale bars: 50 um.

To explore the molecular mechanisms underlying the observed electrophysiological defects in *Etv1* cKO mice, we probed whether rapid conduction gene networks were perturbed in these hearts. To test whether these genes were altered due to cardiomyocyte loss of ETV1, we performed quantitative RT-PCR (qPCR) on FACS-purified VMs, AMs, and PCs from 10–12-week-old *Etv1* cKO and WT hearts in a *Cntn2-*EGFP background. *Nkx2–5*, *Gja5*, and *Scn5a* expression was significantly reduced in *Etv1 c*KO AMs and PCs compared to WT controls while unaltered in VMs where ETV1 is not expressed (Fig. 3A, Supplemental Table 3). In agreement, immunoblots demonstrated reduction of NKX2–5, Cx40, and Na_V_1.5 protein in *Etv1* cKO atrial lysates compared to WT controls (NKX2–5, 57% ± 9%; Cx40, 48% ± 16%; and Na_V_1.5, 64% ± 5%; *P* < 0.05; cKO vs. WT) (Fig. 3B,C, Supplemental Table 4). Regional protein expression was further evaluated using immunofluorescence to identify altered expression of fast conduction genes in *Etv1* cKO and WT cardiac regions. (Fig. 3D,E) The enrichment of NKX2–5, Cx40, and Na_V_1.5 in WT AMs (Fig. 3D) and the proximal HPS (Fig. 3E) were reduced to ventricular levels with cardiomyocyte loss of ETV1.

### Hypoplasia of the His-Purkinje system in cardiomyocyte-specific Etv1 knockout mice

**Figure 4:**
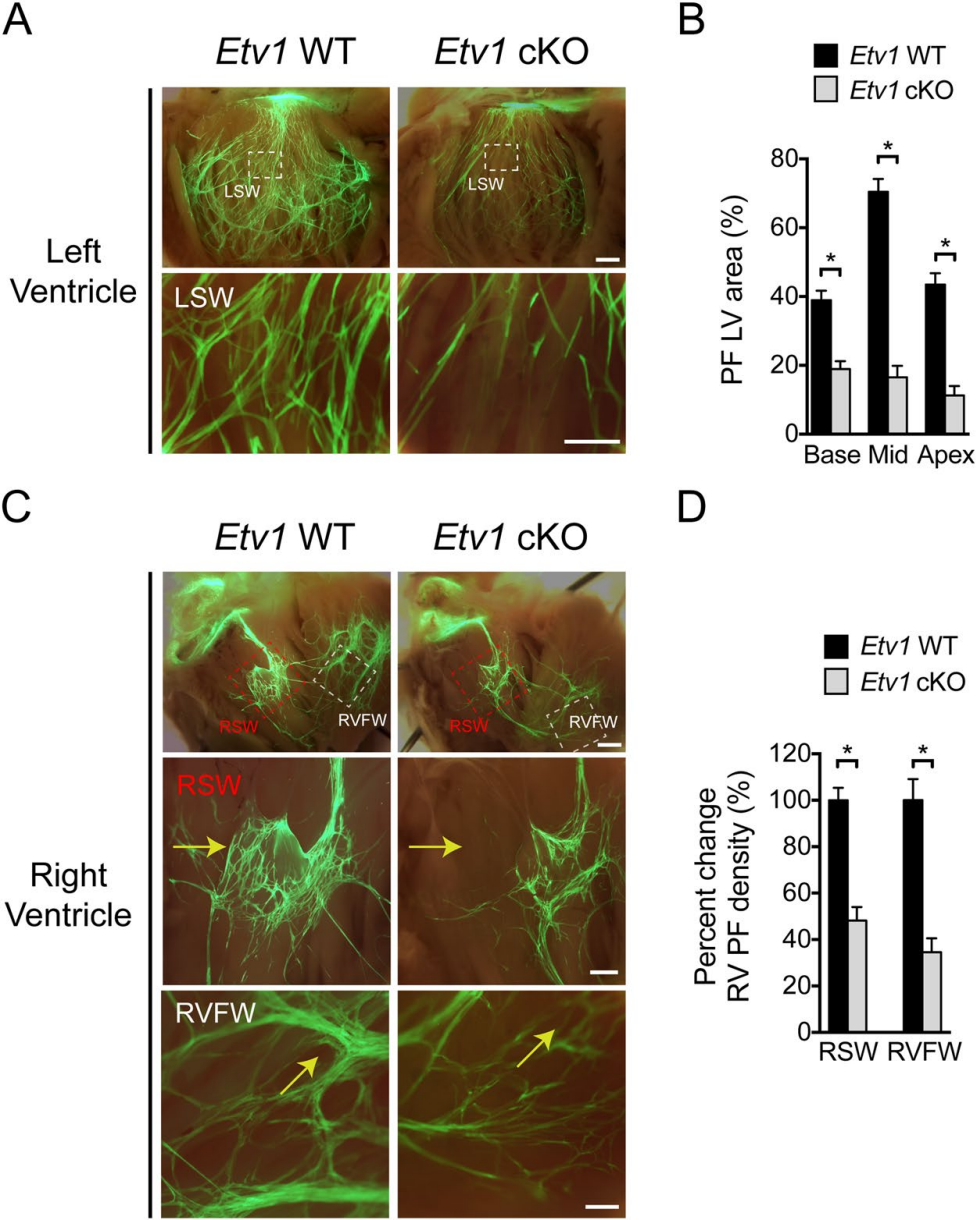
ETV1 is required for normal development of the His-Purkinje system (HPS). *Etv1*^*flox/flox*^ mice backcrossed with *Cntn2*-EGFP were used to visualize the left and right HPS. (**A**) Representative images of the left ventricle (LV) HPS of 10-week-old *Etv1* WT (*Etv1*^*flox/flox*^) *Cntn2-*EGFP and *Etv1* cKO (*Etv1*^*flox/flox*^, *Myh6-*Cre) *Cntn2-*EGFP hearts. Lower row is a high-magnification image of the white dashed boxes in the top row. (**B**) Quantification of EGFP^+^ Purkinje cell density (base, mid and apex) in *Etv1* WT and *Etv1* cKO hearts. Percentage of EGFP^+^ Purkinje cell area was significantly reduced on the left ventricular septal wall (LSW) in *Etv1* cKO hearts versus control (n = 3). (**C**) Representative images of the right ventricle (RV) HPS of 10-week-old *Etv1* WT *Cntn2-*EGFP and *Etv1* cKO *Cntn2-*EGFP hearts. Middle and bottom rows are high-magnification views of red and white dashed boxes outlined in the top row. Yellow arrows highlight regional loss of HPS cells. (**D**) Percentage of EGFP^+^ Purkinje cell area was significantly reduced on the RV septum wall (RSW) and RV free wall (RVFW) in *Etv1* cKO *Cntn2-*EGFP hearts versus control (n = 3). Data represent mean ± SEM. *P < 0.05, 2-tailed Student’s t test. Scale bars: 1 mm (top) (**A,B**), 200um (middle, bottom) (**A,B**).

Cardiomyocyte loss of ETV1 resulted in diminished expression of *Nkx2–5* in AM and PCs, suggesting that *Etv1* cKO mice might have an underlying HPS structural deficit that phenocopies defects in *Nkx2–5* heterozygous mice. To further explore the mechanism underlying conduction deficits in *Etv1* cKO, we used the *Cntn2-*EGFP reporter line to visualize the morphology of the adult left and right HPS using whole mount techniques. In these mice, cardiomyocyte loss of ETV1 resulted in a hypomorphic left and right HPS mirroring the defects observed in *Nkx2–5* heterozygous mice (Fig. 4A,C)^22^. Regional analysis of the left and right HPS showed a significant reduction of PC density in all regions of the heart (Fig. 4B,D). Taken together, we conclude that ETV1-dependent signaling pathways in developing cardiomyocytes play a critical role in the development of the mature HPS.

### Single-cell electrophysiology analysis reveals homogenization of sodium channel properties between Etv1-null ventricular, atrial, and Purkinje cardiomyocytes

**Figure 5:**
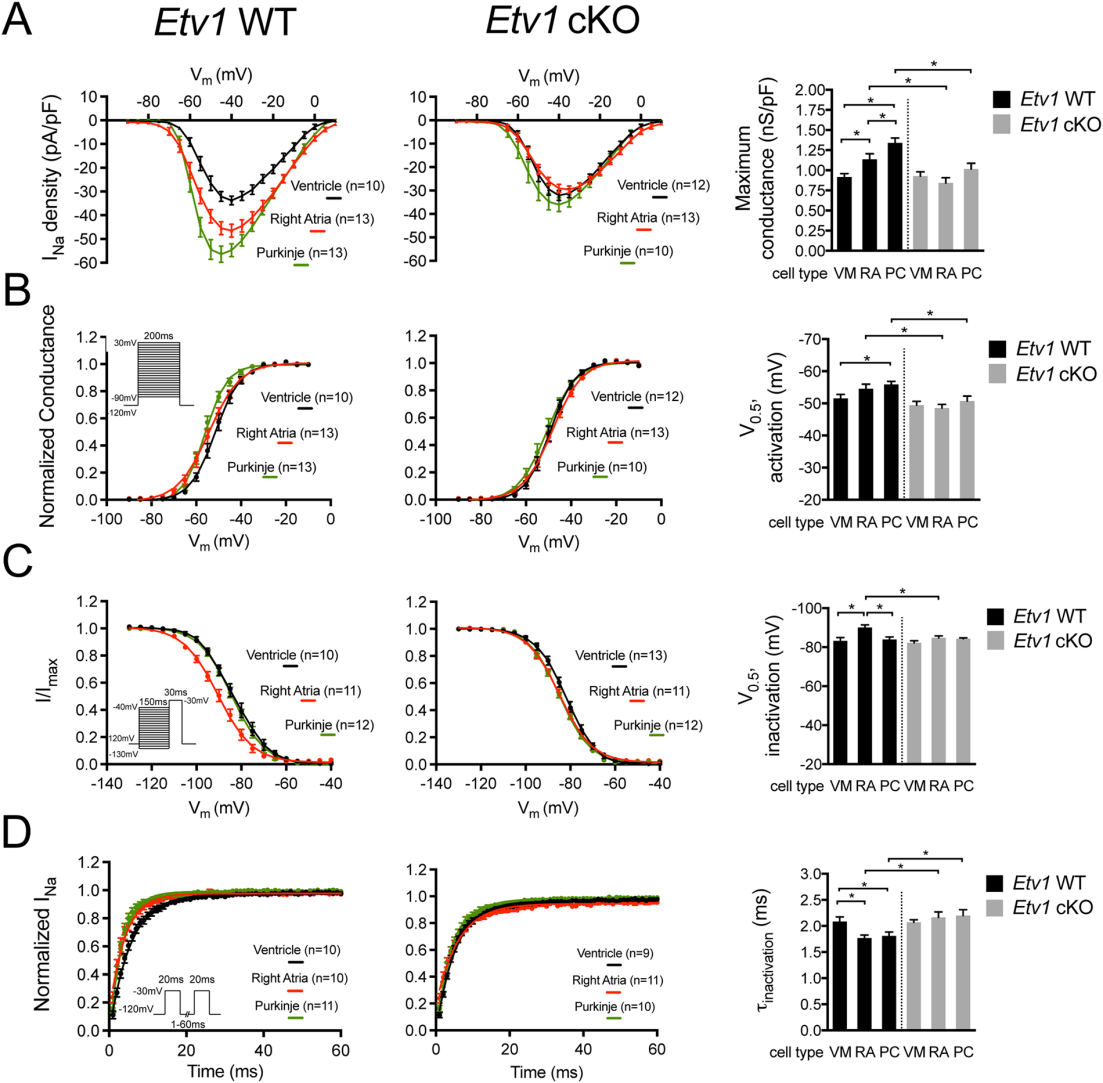
ETV1 regulates the diversity of sodium channel biophysical properties between ventricular, atrial, and Purkinje myocytes. Whole-cell patch clamp data from dissociated cardiomyocytes (ventricular, right atrial, Purkinje myocytes) using 10–12 week-old *Etv1* WT (*Etv1*^*flox/flox*^) and *Etv1* cKO (*Etv1*^*flox/flox*^, *Myh6-*Cre) mice in a *Cntn2*-EGFP background (n = 4). (**A**) Comparison of sodium current–voltage (I–V) relationship. Maximum conductance was calculated to assess significant differences among experimental groups. (**B**) Voltage dependence of steady-state activation. Voltage at half activation (V_0.5_, activation) was calculated to assess significant differences among experimental groups. (**C**) Voltage dependence of steady-state inactivation. Voltage at half inactivation (V_0.5_, inactivation) was calculated to assess significant differences among experimental groups. (**D**) Time course of recovery from inactivation. Tau of recovery (τ_recovery_) was calculated to assess significant differences among experimental groups. Number of cells analyzed per cell type (ventricle, right atria, Purkinje) included in each graph legend. Patch clamp protocol diagrams are included for each endpoint. Data represent mean ± SEM. *P < 0.05, 1-way ANOVA.

Given that *Etv1* cKO hearts show a significant reduction of *Scn5a* in AMs and PCs, we investigated whether cardiomyocyte-specific loss of ETV1 would influence sodium current properties in dissociated adult myocytes. We recorded sodium currents from voltage clamped 10–12-week-old *Etv1* WT and cKO dissociated VMs, AMs, and PCs. In agreement with previous studies^59,60^, voltage step commands elicited the greatest sodium current densities in WT PCs followed by AM and lastly VMs (maximum conductance: 1.34 ± 0.06 nS/pF, 1.14 ± 0.07 nS/pF, 0.92 ± 0.04 nS/pF, respectively) (Fig. 5A, Supplemental Tables 5 and 6). Sodium channels in WT right AMs and PCs underwent steady-state activation at more negative potentials compared to VMs (Fig. 5B, Supplemental Tables 5 and 7). Sodium channels in WT right AMs underwent steady-state inactivation at more negative potentials compared to VMs and PCs (Fig. 5C, Supplemental Tables 5 and 8). Sodium channel recovery from inactivation was faster in right AMs and PCs compared to VMs (Fig. 5D, Supplemental Tables 5 and 9). Cardiomyocyte loss of ETV1 altered the sodium current biophysical properties of AMs and PCs to resemble the sodium current identity of VMs (Fig. 5A–D, Supplemental Tables 5–8).

###  ETV1 overexpression reprograms neonatal ventricular cardiomyocytes towards a His-Purkinje cell gene signature

Next, we examined whether forced expression of ETV1 in P1 neonatal rat ventricular myocytes (NRVMs), which normally lack ETV1, was sufficient to enrich for rapid conduction gene networks. NRVMs monolayers were infected with an adenovirus that co-expresses mouse ETV1 and GFP (Ad-Etv1-EGFP), or GFP alone (Ad-EGFP). Four days post-transduction, cells were collected for either RNA or protein level analysis (Supplemental Fig. 4A). NRVM transduced with Ad-Etv1-EGFP results in a ~49 fold increase in *Etv1* quantified by qPCR (Supplemental Fig. 4B) and robust nuclear expression of ETV1 visualized by Immunofluorescence (Supplemental Fig. 4C).

**Figure 6:**
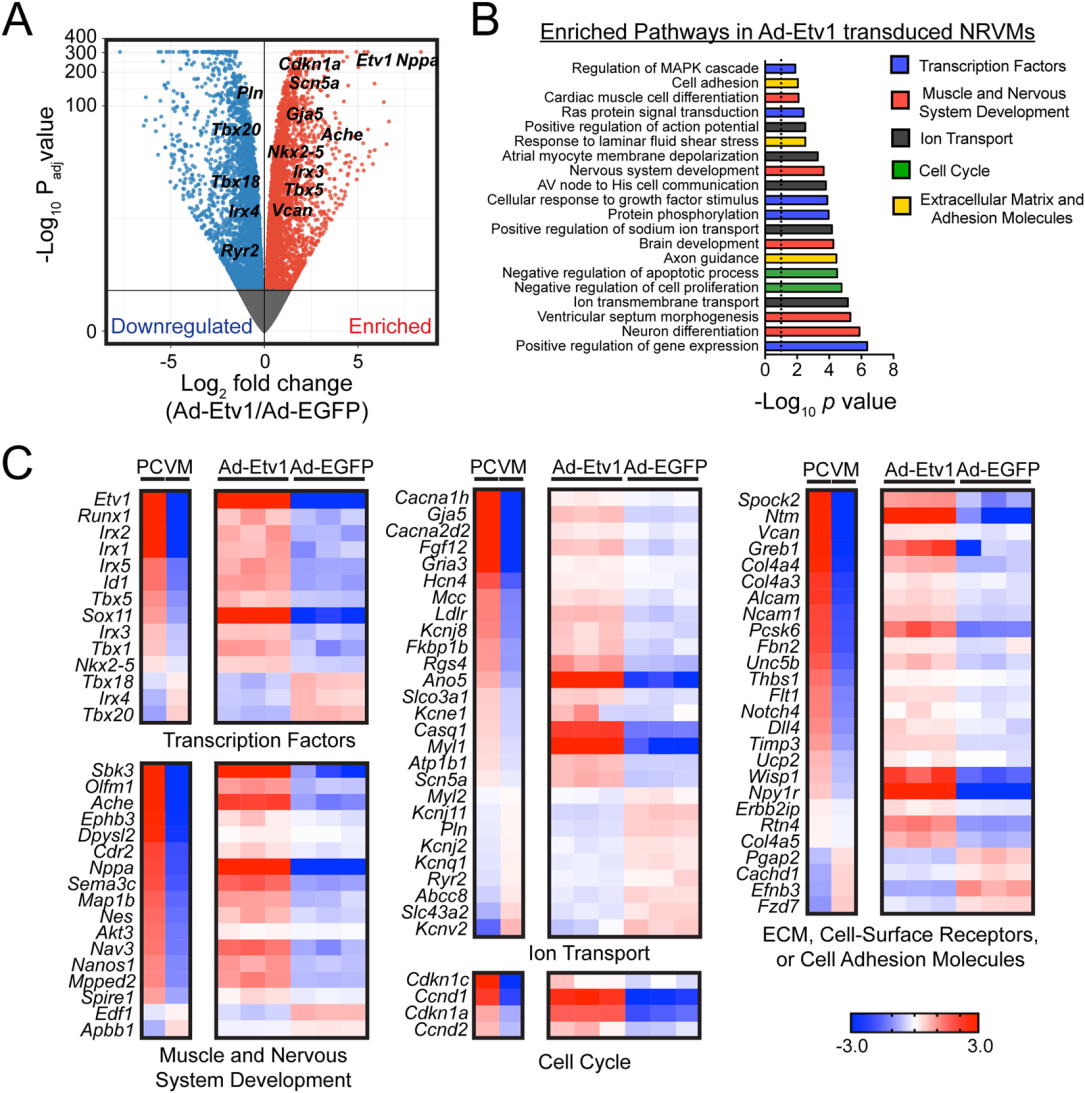
ETV1-transduced neonatal rat ventricular myocytes (NRVMs) upregulates a His-Purkinje system gene signature. (**A**) Volcano plot of relative transcript expression from NRVMs transduced with either Ad-Etv1-EGFP or Ad-EGFP. RNA-sequencing (RNA-seq) comparison revealed a total of 9,236 differentially expressed genes (normalized counts ≥ 5, p_adj_ < 0.05). All significantly different genes (p_adj_ < 0.05) are labeled blue (downregulated) or red (enriched) and all nonsignificantly different transcripts labeled in gray. Of these there were 4,696 upregulated and 4,540 downregulated genes in Ad-Etv1-EGFP versus Ad-EGFP transduced NRVMs. (**B**) Functional clustering of upregulated genes in Ad-Etv1 transduced NRVMs highlighted significantly enriched ETV1-dependent cellular processes (top 20 non-redundant categories are shown). Pathways are color coded to represent genes clustered into functional classes for heat maps in C. (**C**) Comparative RNA-seq between 21-day-old (P21) wild-type mouse FACS-purified Purkinje cell (PC)/ventricular myocytes (VM) and Ad-Etv1-EGFP/Ad-EGFP transduced NRVMs. Heat map representation of 88 genes differentially expressed in Ad-Etv1-EGFP versus Ad-EGFP transduced NRVMs (n = 3) plotted adjacent to average fold change expression in PCs and VMs. Genes clustered into functional groups demonstrate that ETV1 regulates a PC transcriptome in neonatal cardiomyocytes.

To comprehensively explore the GRNs controlled by ETV1, we performed RNA-seq transcriptional profiling on NRVMs transduced with either Ad-Etv1-EGFP or Ad-EGFP. We found a majority of genes were significantly different (9,236 genes, 62%) from a total of 14,813 genes tested with detectible counts (normalized counts > 5). Indeed, rapid conduction genes *Nkx2–5* (1.5 fold, *P*_*adj*_ = 5.4E-27), *Gja5* (2.6 fold, *P*_*adj*_ = 1.8E-87), and *Scn5a* (3 fold, *P*_*adj*_ = 5.0E-140) were all significantly enriched with ectopic ETV1 expression along with numerous other genes identified as critical markers of trabecular/PCs identity, including *Nppa*, *Cdkn1a*, *Ache*, *Irx3*, *Tbx5*, *Kcne1*, and *Vcan* (Fig. 6A)^51,61–64^. In addition, genes associated with nodal or VM identity including *Irx4*^65^, *Tbx18*^66^, *Tbx20*^67^, *Ryr2*^68^, and *Pln*^69^ were all significantly diminished with ectopic ETV1 expression (Fig. 6A). To classify the GRNs altered with ETV1 overexpression, we performed KEGG functional network analysis on the 4,696 upregulated genes and found that many of these genes belonged to nervous system development pathways (Fig. 6B). A majority of these functional pathways including neuron differentiation, axon guidance, neuron development, and cell adhesion were consistent with known transcriptional targets of ETV1 in nervous system development^70^. Indeed, many of these functional networks were enriched in PCs, suggesting that ETV1 plays a key role in endowing cardiac PCs with their rapidly conducting, neuronal-like identity (Fig. 6B, Supplemental Fig. 2B)^47^.

To compare the gene expression profile between endogenous PCs and VMs (Supplemental Fig. 2A) with NRVMs transduced with either Ad-Etv1-EGFP or Ad-GFP (Fig. 6A), heat maps were populated with genes identified via KEGG analysis and clustered into functional groups including TFs, muscle and nervous system development, ion transport, cell cycle, and ECM, cell-surface receptor, or cell adhesion molecules. Heat maps of average log2 fold change values were plotted for PCs and VMs while individual log2 fold change values were plotted for Ad-Etv1-EGFP or Ad-GFP transduced NRVMs demonstrating reproducibility between replicates (Fig. 6C). The gene expression profile of these 88 transcripts revealed a high level of similarity in PC gene expression patterns compared to ETV1-transduced NRVMs. This data establishes that ETV1-directed GRNs are an important component of the PC transcriptome.

**Figure 7:**
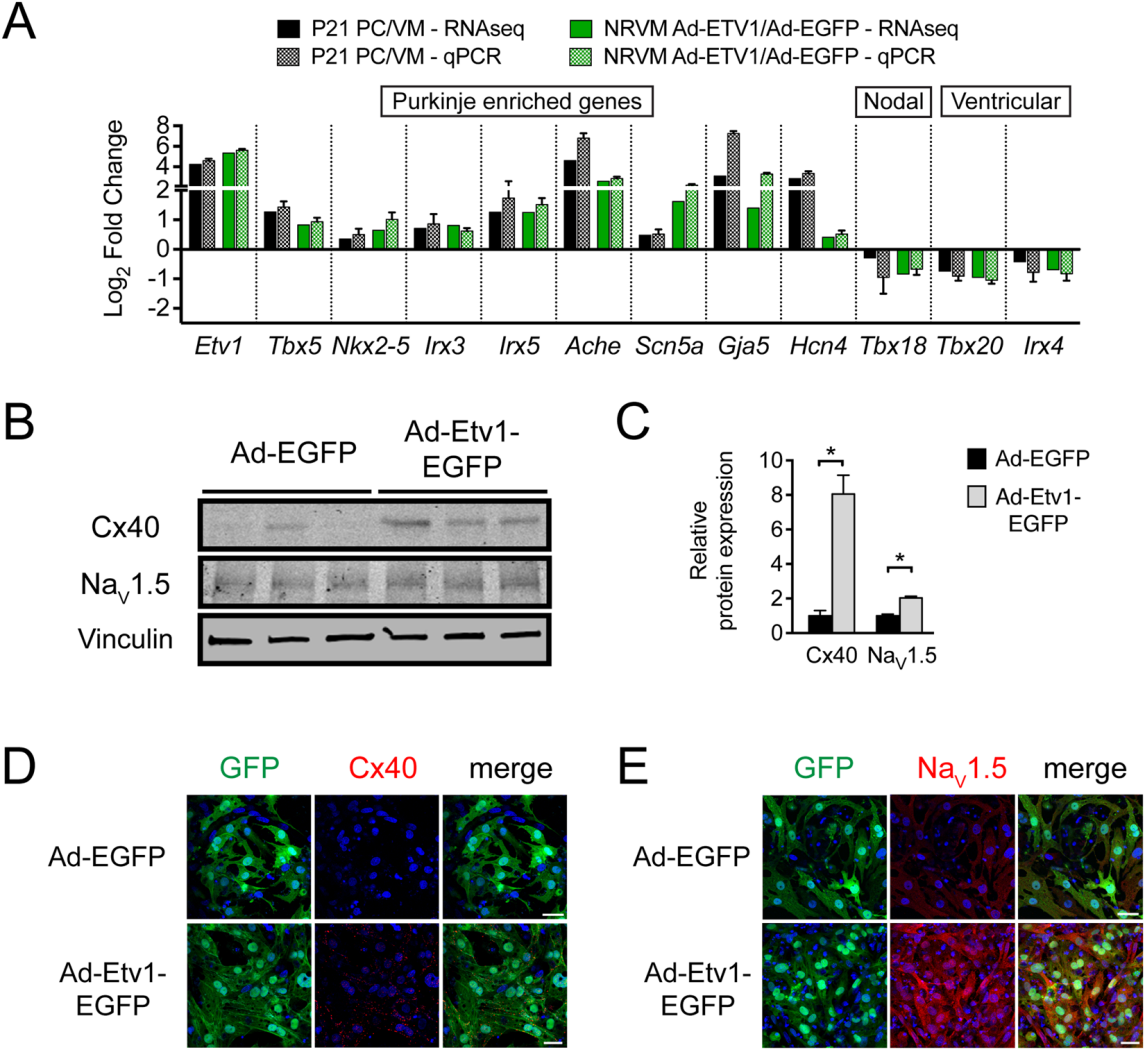
ETV1 overexpression leads to marked enrichment of fast conduction genes in postnatal ventricular myocytes (VMs). (**A**) Quantitative RT-PCR (qPCR) validation of RNA sequencing (RNA-seq) gene expression data comparing known cardiac conduction associated genes from endogenous Purkinje cells (PCs) and VMs versus Ad-Etv1-EGFP or Ad-EGFP transduced neonatal rat ventricular myocytes (NRVMs). Log_2_ fold change displayed relative to VM levels (P21 PC/VM, n = 7,10 respectively) or Ad-EGFP levels (NRVM Ad-Etv1-EGFP/Ad-EGFP, n = 3) (Supplemental Table 10). (**B**) Western blots of Ad-EGFP and Ad-Etv1-EGFP transduced NRVM lysates detecting Cx40, Na_V_1.5, and Vinculin (loading control). (**C**) Protein level densitometric quantification (normalized to vinculin), displayed relative to Ad-EGFP (n = 5). (**D**,**E**) Immunofluorescence staining of (**D**) Cx40 and (**E**) Na_V_1.5 in Ad-Etv1-EGFP and Ad-EGFP transduced NRVMs. ETV1-transduced cells displayed ectopic expression of Cx40 and marked enrichment of Na_V_1.5 compared to Ad-GFP-transduced NRVMs. Nuclei were co-stained with DAPI. Data represent mean ± SEM. *P < 0.05, 2-tailed Student’s t test. Scale bars: 50 um.

We next confirmed select ETV1-regulated genes critical for cardiac conduction by qPCR, including PC-enriched *Etv1*, *Tbx5*, *Nkx2–5*, *Irx3*, *Irx5*, *Ache*, *Scn5a*, *Gja5*, *Hcn4*; Nodal *Tbx18*; and Ventricular *Tbx20*, *Irx4* (Fig. 7A, Supplemental Table 10). Forced expression of ETV1 was sufficient to promote a PC identity in postnatal VMs. We further confirmed that ETV1 was sufficient for protein level enrichment of the gap junction Cx40 and the cardiac sodium channel Na_V_1.5, both necessary for rapid conduction. Immunoblots of NRVMs expressing ETV1 demonstrated a substantial increase in Cx40 protein expression (~8 fold increase) as well as an increase in Na_V_1.5 expression (~2 fold increase) compared to control (Fig. 7B,C). Protein expression changes were further evaluated using immunofluorescence to detect expression of Cx40 and Na_V_1.5 in Ad-Etv1-EGFP or Ad-EGFP transduced NRVMs (Fig. 7D,E). Cx40 was ectopically detected in VMs with transduction of ETV1 (Fig. 7D). Moreover, the expression of Na_V_1.5 was markedly enriched in VMs expressing ETV1 (Fig. 7E).

### ETV1 induces expression of rapid conduction genes and Purkinje-like sodium currents on human induced pluripotent stem cell-derived cardiomyocytes

**Figure 8:**
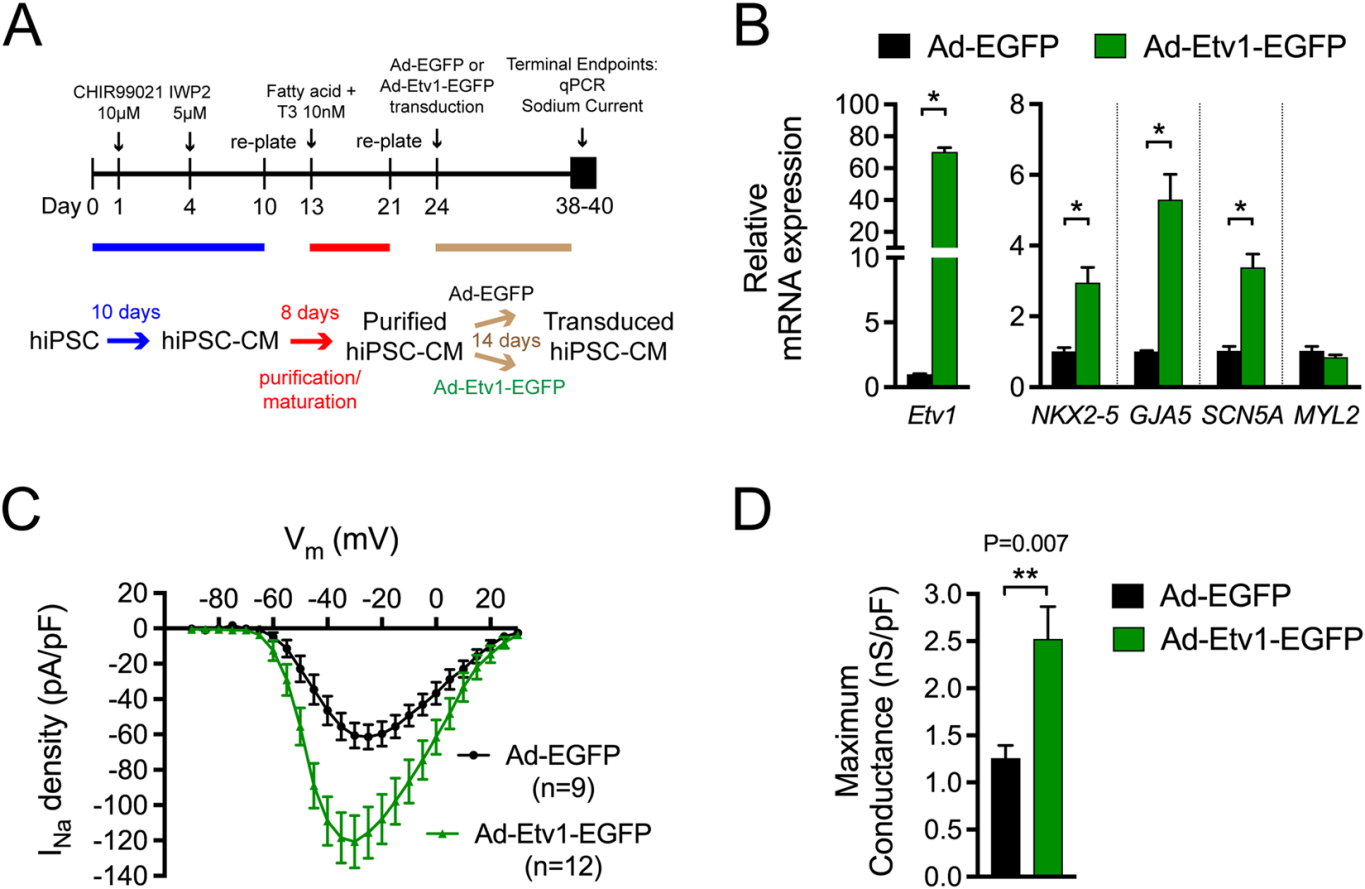
Activation of ETV1 in human induced pluripotent stem cells-derived cardiomyocytes (hiPSC-CMs) leads to increased expression of rapid conduction genes and sodium current. (**A**) Schematic representation of hiPSC-CM generation and maturation (day 0–21), transduction of Ad-Etv1-EGFP or Ad-EGFP (day 24), and timepoint for experimentation (day 38–40). (**B**) Quantitative RT-PCR analysis of *Etv1*, *NKX2–5*, *GJA5*, *SCN5A*, and *MYL2* in hiPSC-CM transduced with either Ad-Etv1-EGFP or Ad-EGFP (n = 4). (**C**) Whole-cell patch clamp was performed on Ad-Etv1-EGFP (n = 12) or Ad-EGFP (n = 9) transduced hiPSC-CMs. Sodium current–voltage (I–V) relationship comparison. (**D**) hiPSC-CM Na_V_ peak conductance (gNa_V_-peak). gNa_V_-peak following −120 mV to −35 mV depolarization step was measured for Ad-Etv1-EGFP (n = 12) or Ad-EGFP (n = 9) transduced hiPSC-CMs. Data represent mean ± SEM. *P < 0.05, 2-tailed Student’s t test.

To assess whether ETV1 has a conserved function in human HPS specification, we investigated whether ectopic ETV1 induction in hiPSC-CMs would convert these cells toward a HPS lineage. Using a protocol previously validated for the generation of purified and mature hiPSC-CMs^71^, we infected hiPSC-CM monolayers with Ad-Etv1-EGFP or Ad-EGFP. Two weeks post-transduction, cells were collected for either RNA or cellular electrophysiological analysis (Fig. 8A). hiPSC-CMs transduced with Ad-Etv1-EGFP results in a ~70 fold increase in *Etv1* quantified by qPCR (Fig. 8B). Similar to results observed in NRVMs, hiPSC-CMs expressing *Etv1* demonstrated a substantial increase in *NKX2–5* expression (3.0 fold, *P* = 4.6E-3), *GJA5* expression (5.3 fold, *P* = 9.5E-4), and *SCN5A* expression (3.4 fold, *P* = 9.9E-4) compared to control (Fig. 8B, Supplemental Table 11). ETV1 was able to dictate rapid conduction GRNs in human cardiomyocytes.

To probe whether these changes in gene expression altered sodium current properties, we recorded sodium currents from voltage clamped transduced hiPSC-CMs. Voltage step commands elicited larger transient sodium current densities in *Etv1* transduced hiPSC-CMs (n = 12) versus *GFP* transduced hiPSC-CMs (n = 9) (Fig. 8C). Peak currents in *Etv1* transduced hiPSC-CMs were double compared to controls (maximum conductance: 2.52 ± 0.33 nS/pF, 1.26 ± 0.014 nS/pF, respectively) (Fig. 8C,D, Supplemental Table 12). Sodium channels in *Etv1* transduced hiPSC-CMs trended to undergo steady-state activation at more negative potentials compared to control (Supplemental Fig. 5A, Supplemental Table 12). No difference was observed in sodium channel steady-state inactivation in transduced hiPSC-CMs (Supplemental Fig. 5B, Supplemental Table 12). Taken together, *Etv1* overexpression was able to transform the sodium current properties of hiPSC-CMs towards a PC-like phenotype.

## Discussion

Rapid conduction through the atria and specialized HPS enables coordinated activation and contraction of the four-chamber heart. Our studies reinforce ETV1 as a key regulatory factor that controls rapid conduction cardiac GRNs. Using cardiomyocyte-specific loss of function and viral transduction strategies, we found that ETV1 positively regulated gene networks necessary for active (*Scn5a*) and passive (*Gja5*) conductance along with structural (*Nkx2–5*) determinants of the mature HPS. As a consequence, cardiomyocyte loss of ETV1 significantly slows conduction in atrial and His-Purkinje myocytes and produces severe specification defects of the adult HPS. When placed in the context of previous work^19–21^, endocardially-derived NRG1 signaling establishes progenitor HPS cells and simultaneously activates ETV1 signaling networks to create rapid conduction regions. Our study now links this established upstream signaling network with downstream cardiomyocyte-specific nuclear responses that occur to create distinct electrical networks in the adult heart.

Purkinje and VMs share a common ventricular progenitor^12^. Their transcriptional signature begins to diverge with the inception of NRG1 signaling gradients creating trabecular and compact zones within the embryonic heart^20^. To define the transcriptional landscape of rapidly conducting PCs, we probed transcriptional differences between PCs and VMs by RNA-seq. We found that *Etv1* is the most differentially expressed TF between PC and VMs. In addition, we identified that *Etv1* is one of the most abundant expressed TFs in PCs. Confirming this approach, we also show *Tbx5*, another established HPS regulator required for maintenance of normal cardiac rhythm, to be highly abundant and enriched within HPS^26,27,72^. We believe this newest dataset is the benchmark for defining the transcriptional identity of endogenous murine cardiac PCs for comparative investigation.

To verify the specificity of *Etv1* expression within the heart, we analyzed a BAC transgenic mouse, *Etv1-*EGFP. This reporter mouse was originally developed to identify and study neuronal cell types;^56,73^ we speculated it would also be an effective tool to study the HPS with the added benefit of not altering *Etv1* gene dosage. In line with our previous description of the *Etv1-nlz* reporter in the heart^21^, *Etv1*-EGFP expression was observed throughout the HPS at all stages of cardiogenesis and the mature heart. GFP expression was also detected in the PAM in the developing and mature heart. This suggests that ETV1-dependent GRNs establish early in development and are engaged in atrial and trabecular myocytes. During the transition from precursor trabecular myocytes to a Purkinje lineage, ETV1 continues to be highly expressed in cells destined to populate the post mitotic HPS, and persistent ETV1 activity may be required to promote conduction health. Moreover, the commonality of ETV1 expression in PAM and HPS cells reveal a mechanistic understanding of the shared GRNs and electrical properties that govern these fast conducting, heterogeneous cell types.

ETV1 is expressed within many neuronal cell types and plays an important role in their specification^41,43,45,57,70,74^. Our initial germline knockout studies were not able to address whether neuronal influences contributed to the observed cardiac conduction phenotype. It is well recognized that neuronal cell types, particularly neural crest cells, are required for normal heart development^75^. Cardiomyocyte loss of ETV1 circumvents the adolescent lethality observed in *Etv1* germline KO mice, enabling electrophysiological assessment of the adult heart. We found that *Etv1* cKO mutant hearts resemble *Etv1* germline null hearts in virtually all aspects of functional, molecular, and cellular electrophysiological phenotype. Conduction defects including P wave and QRS interval prolongation in *Etv1* cKO hearts arose from a combination of hypoplastic HPS creating current-to-load mismatch, decrease in junctional conductance, and alterations in sodium channel density and gating; all present in *Etv1* germline KO heart. A key point of distinction between *Etv1* germline KO hearts and *Etv1* cKO hearts is that *Etv1* cKO hearts had a normal PR duration, suggesting normal AV nodal function^21^. This difference could suggest a neuronal component to AV nodal development, an effect of ETV1 on autonomic innvervation of the AVN, or a lack of *Etv1* deletion in E9.0 cardiomyocytes that populate the developing AVN. This observation supports a need for future experiments focused on elucidating the role of ETV1 in AV nodal development and/or autonomic innervation. Neuronal-restricted gene targeting will prove important to elucidate these potential mechanisms.

Our ETV1 transduction study demonstrates that this single TF is sufficient to impart a PC genetic program onto postnatal VMs. Indeed, ETV1 is an established orchestrator of neuronal GRNs and appears to convey a neuronal-like gene signature to cardiac PCs. When compared to our Purkinje genome wide expression profile the transition of ETV1 transduced VMs to a PC identity appears to be of high fidelity. Genes required for rapid conduction and HPS specification were significantly enriched including *Gja5*, *Scn5a*, *Nkx2–5*, *Tbx5*, *Irx3*, and *Irx5*. Moreover, classic markers of the HPS^49^ were also induced in ETV1 transduced VMs including *Ache*, *Kcne1* (MinK), *Hcn4*, and *Cacna1h*. Of note, ectopic ETV1 expression in VMs was not able to enrich mature PC markers including *Pcp4*^47^ and *Cntn2*^53,76^. Since NRVM cultures are only viable up to one week, it will be interesting to see whether *in vivo* transductions that facilitate more prolonged expression of ETV1 could mitigate these shortcomings or if other unknown TFs are required for expression of these terminal markers. Similar to our work, a recent study using IRX3 overexpression in NRVMs demonstrated that IRX3 was able to promote expression of 12 HPS-enriched genes^33^. In their model, IRX3 interacts with NKX2–5/TBX5 in a post-translational TF complex required for proper HPS development and function^33^. Our work extends their findings by demonstrating that ETV1 transcriptionally activates these TFs for appropriate specification of the HPS.

The application of this pathway to hiPSC-CMs suggests a conserved role of ETV1 in defining rapid conduction GRNs across vertebrate species. Human and mouse ETV1 are both encoded by 13 exons, share a highly conserved amino acid sequence (98%), and have identical sequence homology in the DNA binding ETS domain (Supplemental Fig. 6)^77^. Indeed, the conserved function of ETV1 has been probed in dopaminergic neurons where mouse ETV1 was able to rescue the *ast-1* (*Etv1* ortholog) mutant phenotype when expressed in transgenic worms^74^. Our study suggests that mouse ETV1 is able to induce rapid conduction genes and modify sodium current properties resembling a PC-like signature in hiPSC-CM reinforcing a shared mechanism. To our knowledge, ETV1 is the first TF sufficient to modify the transcriptome and sodium current properties of human cardiomyocytes towards a PC-like phenotype. Investigating whether ETV1 alone or combined with other TFs and/or signaling pathways, such as Notch^78^ or NRG1^19^, are able to differentiate adult VMs or pluripotent stem cells into mature His-PCs will be of significant interest.

Current treatment options to improve conduction in failing hearts are limited to artificial pacemakers. While these implantable devices have proved to be clinically effective, they do not address the causal elements of impaired electrical function. Our findings shed light on the cell-type specific molecular mechanisms that orchestrate HPS development and rapid conduction physiology. Moreover, our data suggest that the ETV1-dependent rapid conduction GRN can be modulated for the management of selected human conduction disorders.

## Methods

### Experimental animals and Study design

All procedures and protocols were approved by the Institutional Animal Care and Use Committee of the New York University Langone Health, protocol IA16–01599, and complied with the standards for the care and use of animal subjects as stated in the *Guide for the Care and Use of Laboratory Animals*.

This study was preformed using *Etv1*^*flox/flox*^ mice^46,57^ (provided by Dr. Ping Chi, Memorial Sloan-Kettering Cancer Center NY, NY, USA), *Myh6*-Cre^58^, *Cntn2-*EGFP BAC transgenic^53^, and *Etv1-*EGFP BAC transgenic^56^ mutant mice that have been previously characterized. *Cntn2-*EGFP and *Etv1-*EGFP mice were maintained in the CD1 genetic background. *Etv1*^*flox/flox*^ and *Myh6*-Cre mice were maintained in the C57BL/6 genetic background. For Purkinje cell FACS, HPS morphology imaging/quantification, and cellular electrophysiology, *Etv1*^*flox/flox*^; *Myh6*-Cre mouse line was bred into the *Cntn2-*EGFP background. *Etv1*^*flox/flox*^; *Myh6*-Cre*; Cntn2-*EGFP compound mutant mouse lines were backcrossed greater than 5 generations into the C57BL/6 genetic background. *Etv1*^*flox/flox*^ mice were used as WT controls. For NRVM isolations, P1 wild type litters in a Sprague-Dawley background were purchased from Charles River Laboratories.

### NRVM isolation

NRVMs were isolated from P1 wild type male and female rats using the Miltenyi Biotec neonatal heart dissociation kit (Miltenyi Biotec, 130–098–373) with a gentleMACS octo dissociator (Miltenyi Biotec, 130–096–427) according to the manufacture’s protocol. P1 NRVM heart lysates were purified using Miltenyi Biotec rat neonatal cardiomyocyte isolation kit (Miltenyi Biotec, 130–105–420) according to the manufacture’s protocol. The final cell suspension was collected, counted, and plated as confluent monolayers on fibronectin-coated (25 μg/mL) plastic dishes. 24 hours post-plating in DMEM plus 10% FBS plus supplements (3 mM pyruvic acid, 2 g/l BSA, 0.5 mg/ml primocin, 15 mM HEPES, 4 μg/ml transferrin, 0.7 ng/ml sodium selenite, 5 μg/ml linoleic acid, 10 μM ascorbic acid), cells were cultured in serum-free DMEM with supplements as previously described^21^. The final myocyte cultures contained purified cardiomyocytes at a density of 3 × 10^6^ cells per 35-mm dish. Viral transductions were performed during the media change at 24 hours after plating. Cardiomyocytes were used for either transcript or protein level analysis four days post-transduction.

### Human induced pluripotent stem cell lines

NCRM1 iPSC line from Codex BioSolutions Inc. (MD, USA) were plated on Geltrex LDEV-Free Reduced Growth Factor Basement Membrane Matrix (Gibco, A1413202) coated plates and cultured with Essential 8 Medium (Gibco, A1517001). The differentiation and maturation protocol has been previously published^71^. Briefly, day one hiPSC were treated with small molecule CHIR99021 (Tocris, 4423, final concentration 10 μM) in the RPMI-BSA medium [RPMI 1640 Medium (HyClone, SH30027.01) supplemented with 213 μg/ml AA2P (l-ascorbic acid 2-phosphate magnesium) (A8960, Sigma) and 0.1% bovine serum albumin (BSA) (A1470, Sigma)] for 24 h followed by RPMI-BSA medium replacement. On differentiation day four, cells were treated with IWP2 (Tocris, 3533, final concentration 5 μM) in RPMI-BSA medium. After 48 h, media was replaced with RPMI-BSA medium. During maturation and purification, differentiated hiPSC-cardiomyocytes were treated with metabolic selection medium (fatty acid + T_3_). Fatty acid medium was prepared as DMEM Medium (No Glucose) supplemented with 0.1% BSA (Sigma, A1470) and 1 × Linoleic Acid-Oleic Acid-Albumin (Sigma, L9655). Fatty acid + T_3_ medium was fatty acid medium supplemented with T_3_ (Acros Organics, 437260010, final concentration 10 nM) for 8 days. RPMI 1640 Medium supplemented with 3% KnockOut Serum Replacement (Gibco, 10828–028, the routine medium) was used to culture differentiated cardiomyocytes. Medium was changed every two days unless specified.

### Adenovirus reagents

A monolayer of NRVMs or iPSC-CMs was transduced with either Ad-Etv1-EGFP (*Etv1* vector; multiplicity of infection 5:1; Vector Biolabs) or Ad-EGFP (control vector; multiplicity of infection 5:1; Vector Biolabs) 24 hours post cell isolation (NRVMs) or 24 days post hiPSC differentiation.

### Antibody reagents

Immunofluorescent antibodies used in this study were all previously described^21^. The antibody target, dilution, species, company, and product number are the following: NKX2–5, 1:100 (rabbit, Abcam, ab91196); ETV1, 1:100 (rabbit, Abcam, ab36788); Na_V_1.5, 1:50 (rabbit, Alomone Labs, ASC-005); Cx40, 1:250 (rabbit, Alpha Diagnostic, Cx40A); CNTN2, 1:40 (goat, R&D Systems, AF4439); GFP, 1:100 (rabbit, Abcam, ab290). Secondary antibodies were donkey anti-rabbit, 1:500 (Santa Cruz Biotechnology, sc-2784); donkey anti-goat, 1:500 (Santa Cruz Biotechnology, sc-2024); and donkey anti-mouse, 1:500 (Santa Cruz Biotechnology, sc-2099). Western blot primary antibodies were [target, dilution (species, company, product number)] Nkx2–5, 1:1,000 (mouse, Abcam, ab91196); ETV1, 1:200 (goat, Santa Cruz Biotechnology, sc-1953); Na_V_1.5, 1:500 (rabbit, Alomone Labs, ASC-005); Cx40, 1:1,000 (rabbit, Alpha Diagnostic, Cx40A); and vinculin, 1:5,000 (mouse, Abcam, ab11194). Secondary antibodies were goat anti-rabbit, 1:15,000 (LI-COR, 926–32211); goat anti-mouse, 1:15,000 (LI-COR, 926–32220); and donkey anti-goat, 1:15,000 (LI-COR, 926–32214).

### Immunohistochemistry

Embryonic and adult hearts were removed and either emersion or retrograde perfusion-fixed, respectively, and stored in 4% paraformaldehyde overnight (adult) or 2 hours (embryonic) at 4 °C. Samples were then processed as previously described^21^.

### Electrocardiograms

Surface ECGs were acquired in anesthetized mice (2% isoflurane) by using subcutaneous needle electrodes attached in a 3-limb configuration, as previously described^21,79^. Parameters measured from the ECG data included P wave duration, PR interval, QRS duration, and QT interval. The P wave duration included both the first positive and negative deflections from baseline. QRS duration was measured from the initial deflection from baseline after the P wave until the return to baseline prior to the T wave peak. PR interval was measured from the start of the P wave to the beginning of the QRS complex. RSR’ was defined as two upward deflections above baseline resulting from a single ventricular depolarization. The R wave is the first upward deflection in the QRS complex. The S component is the first downward deflection. The R’ wave is a second upward deflection in the QRS complex prior to the T wave peak.

### Cardiomyocyte enzymatic dissociation for whole-cell patch clamp and FACS purification

Cardiomyocytes were isolated from P21 *Cntn2-*EGFP hearts for RNA-seq and 10–12 week old *Etv1*^fl/fl^ *Cntn2-*EGFP hearts for qPCR studies. Hearts were Langendorff-perfused and enzymatically digested as previously described^80^. Myocyte FACS-based purification was performed as previously described^21,81^. Cardiomyocytes were purified based on their mitochondrial content with the incubation of the mitochondrial dye tetramethylrhodamine methyl ester perchlorate (TMRM; Invitrogen). 50 nM TMRM was added to the enzymatic digestion buffer for the Langendorff-perfusion. Cell suspensions were purified by FACS (Beckman Coulter MoFlo, 100 µm nozzle). Purkinje (TMRM^hi^GFP^+^) and ventricular myocytes (TMRM^hi^GFP^–^) fractions were collected for RNA isolation (Supplemental Fig. 1). VMs and PCs for patch clamp experiments were not stained for TMRM. PCs were identified visually via endogenous GFP expression.

### RNA-sequencing and quantitative RT-PCR

Purkinje and Ventricular Myocyte RNA-seq: RNA was isolated from FACS purified Purkinje and Ventricular myocytes by the PicoPure RNA Isolation Kit (Arcturus Engineering). Isolated RNA was amplified and sequencing libraries were prepared using the Trio Low Input RNA Kit (Nugen). NRVM RNA-seq: Total RNAs were extracted using the RNeasy kit (Qiagen). Sequencing libraries were prepared using the TruSeq RNA Library Prep Kit v2 (Illumina). For both sequencing experiments, samples were sequenced 50 bp paired-ended at 10 million to 20 million reads per replicate on an Illumina HiSeq. 2500 instrument. Library preparation and sequencing were performed at New York University School of Medicine Genome Technology Center. All the reads were mapped to the mouse or rat (based on samples genome) reference genome (mm10/Rnor6) using the STAR aligner (v2.5.0c)^82^. Alignments were guided by a Gene Transfer File (GTF, version GRCm38.74) and the mean read insert sizes and their standard deviations were calculated using Picard tools (v.1.126) (http://broadinstitute.github.io/picard/). The read count tables were generated using HTSeq (v0.6.0)^83^ and normalized based on their library size factors using DESeq. 2 (v3.0)^84^ and differential expression analysis was performed. The Read Per Million (RPM) normalized BigWig files were generated using BEDTools (v2.17.0)^85^ and bedGraphToBigWig tool (v4), and downstream statistical analyses and generating plots were performed in R environment (v3.1.1) (http://www.r-project.org/). Gene ontology analysis was performed with DAVID (http://david.abcc.ncifcrf.gov/). Candidate targets were validated with qPCR using primers specific to the gene of interest. (Supplemental Table 13) RNA was reverse-transcribed to cDNA using the SuperScript III First-Strand Synthesis System (Invitrogen). qPCR was performed with a SYBR qPCR Kit (Qiagen) on a StepOne™ Real-Time PCR System (Applied Biosystems). qPCR probes, if available, were purchased from Origene and used according to the manufacturer’s instructions. Remaining qPCR probes were designed or identified and validated from prior literature.

### Whole-cell sodium current (I_Na_) recordings

I_Na_ recordings in isolated cardiomyocytes and hiPSC-CM were conducted in whole-cell configuration at room temperature with voltage step protocols as previously described^21^. As described in published methods^21^, recording pipettes were filled with a solution containing (in mM) NaCl 5, CsF 135, EGTA 10, MgATP 5, HEPES 15, pH 7.2, with CsOH. Cells were maintained in a solution containing (in mM) NaCl 5, CsCl 112.5, TEACl 20, CdCl_2_ 0.1, MgCl_2_ 1, CaCl_2_ 1, HEPES 20, glucose 11, pH 7.4, with CsOH. All recordings were obtained three times to verify reproducible recordings and within 15 minutes after establishing whole-cell configuration using an Axon multiclamp 700B Amplifier coupled to a pClamp system (version 10.2, Axon Instruments).

### Transthoracic echocardiography

Echocardiography was performed on anesthetized (2% isoflurane) mice as previously described^21,86^ using the Vevo 2100 high-resolution ultrasound imaging system (VisualSonics). VisualSonics Vevo 2100 V1.5.0 software was used for all data analysis. Short axis M-mode parameters were measured that include: diastolic and systolic left ventricular anterior and posterior wall thickness. From long axis B-mode measurements, left ventricular end diastolic volume, end systolic volume, stroke volume and ejection fraction were calculated from semi-automated LV area traces

### HPS whole-mount quantification

His-Purkinje system imaging and quantification were conducted using *Etv1*^*flox/flox*^; *Myh6*-Cre; *Cntn2-*EGFP reporter mice as previously described^21^. Hearts were excised, washed in 1% PBS, and retrograde perfusion-fixed with 4% paraformaldehyde and stored overnight. For imaging of the left and right HPS, the left and right free walls were cut open as previously described^21^. Littermate controls (*Etv1*^*flox/flox*^; *Cntn2-*EGFP) were imaged on the same day at comparable magnification, exposure, and light intensity. GFP positive area normalized to total area (as specified) was measured using ImageJ software for the left ventricular septum, right ventricular septum, and right ventricular free wall.

### Statistics

Comparisons were tested using 1-way ANOVA or 2-tailed Student’s *t* test where appropriate. *P* < 0.05 were considered statistically significant. All results were expressed as Mean ± SEM for each group. Mouse ECGs and ECHO data analysis were conducted by 2 operators. Genotypes were blinded until the endpoints were analyzed. Mouse studies were performed prior to genotyping, ensuring blinded observations.

### Data availability

The mouse lines will be made publicly available through a mouse repository. RNA-seq data has been deposited with the NCBI under the Gene Expression Omnibus (GEO) accession number: GSE115061. All other data and materials are available upon corresponding author request.

## Electronic supplementary material

Supplemental Material
