# Supplementary material for: ETV1 activates a rapid conduction transcriptional program in rodent and human cardiomyocytes
Source: Sci Rep. 2018 Jul 2;8:9944. doi: 10.1038/s41598-018-28239-7 (PMC6028599; doi:10.1038/s41598-018-28239-7)
Supplement: Supplementary file 1 — Supplemental Material [file 41598_2018_28239_MOESM1_ESM.pdf]

ETV1 activates a rapid conduction transcriptional program in rodent and human cardiomyocytes

Akshay Shekhar[1], Xianming Lin[1], Bin Lin[1], Fang-Yu Liu[1], Jie Zhang[1], Alireza Khodadadi-Jamayran[2], Aristotelis Tsirigos[2], Lei Bu[1], Glenn I Fishman[1,*], David S Park[1,*]

[1]Leon H. Charney Division of Cardiology, New York University Langone Health, New York, New York, 10016, USA.

[2]Center for Health Informatics and Bioinformatics, New York University Langone Health, New York, New York 10016, USA.

The authors have declared that no conflict of interest exists

*To whom correspondence should be addressed

SUPPLEMENTAL MATERIAL

Supplemental Figures

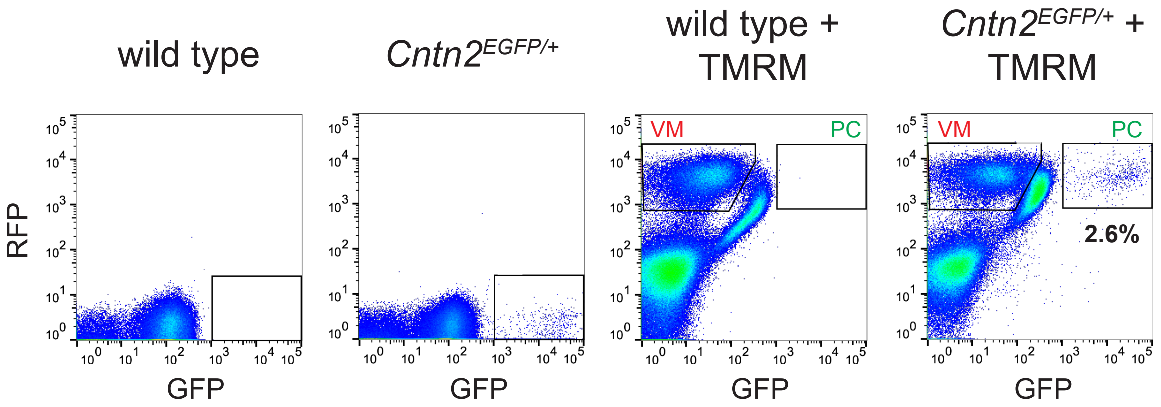

Supplemental Figure 1. Fluorescence-activated cell sorted (FACS) strategy to collect purified mouse Purkinje cells (PC) and ventricular myocytes (VM). Ventricular cell lysates were stained with mitochondrial red dye tetramethylrhodamine methyl ester perchlorate (TMRM) during dissociation to identify the high mitochondrial content cardiomyocyte fraction. FACS dot plots from four groups (wild type, *Cntn2*-EGFP, wild type + TMRM, *Cntn2*-EGFP + TMRM) demonstrate gating strategy to collect purified PCs ($TMRM^{hi}EGFP+$) and VMs ($TMRM^{hi}$ alone).

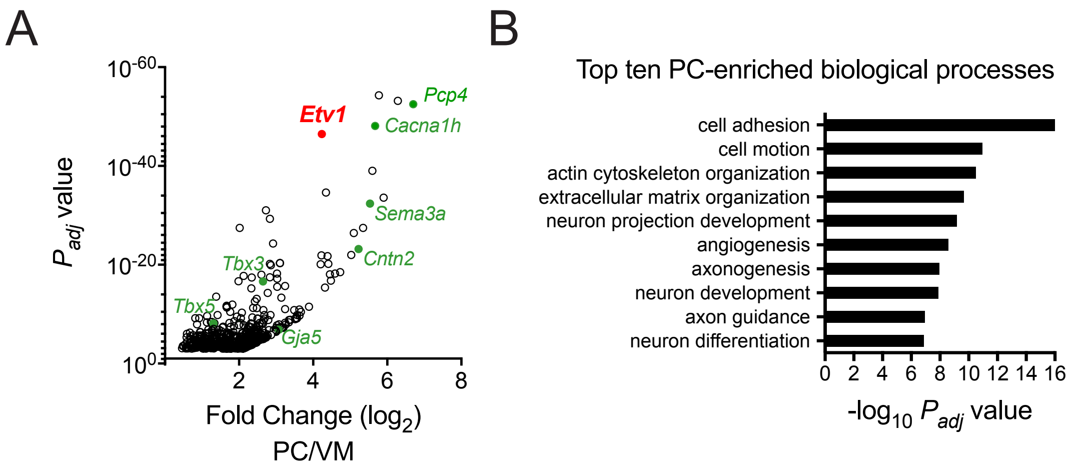

Supplemental Figure 2. RNA sequencing analysis of Purkinje cells (PC) and ventricular myocytes (VM). (A) Expression of PC-enriched genes at postnatal day 21. Log_2 Fold change [PC (n=7) vs VM (n=10)] shown on the x-axis and P_{adj} value shown on the y-axis. Known PC-enriched genes labeled in green validating this FACS-based purification and sequencing approach. (B) GO functional clustering of upregulated genes in PCs. Top 10 statistically significant ($-log_{10}$ P_{adj}), non-redundant categories are shown.

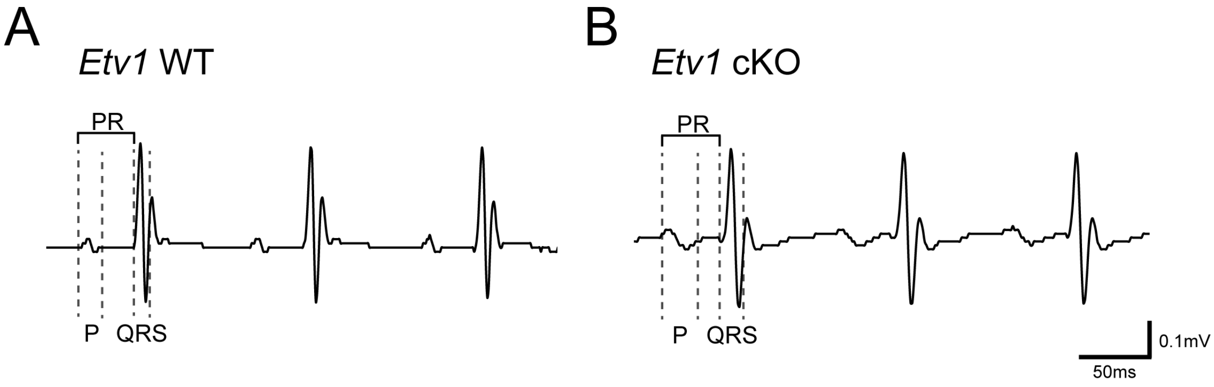

Supplemental Figure 3. Surface ECG traces of 10-week-old *Etv1* WT and *Etv1 c*KO mice without presence of an RsR'. Representative ECGs from (A) *Etv1* WT and (B) *Etv1* cKO mice with markers identifying P, PR, and QRS duration.

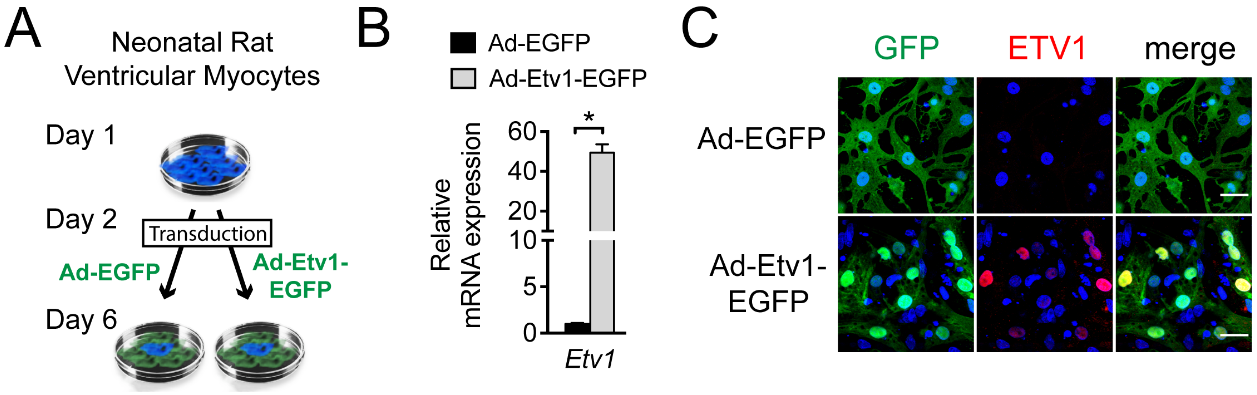

Supplemental Figure 4. ETV1 overexpression strategy in neonatal rat ventricular myocytes (NRVMs). (A) Schematic representation of NRVM isolation (day 1), transduction of Ad-Etv1-EGFP or Ad-EGFP (day 2), and collection for experimentation (day 6). (B) Quantitative RT-PCR analysis confirms marked enrichment of *Etv1* in Ad-Etv1-EGFP transduced NRVMs versus Ad-EGFP (n=3). (C) Immunofluorescence staining of ETV1 in Ad-Etv1-EGFP and Ad-EGFP transduced NRVMs. Ad-EGFP transduced NRVMs did not show evidence of endogenous ETV1 while Ad-ETV1-EGFP transduced NRVMs display nuclear expression of exogenous ETV1 in all GFP^+ cells.

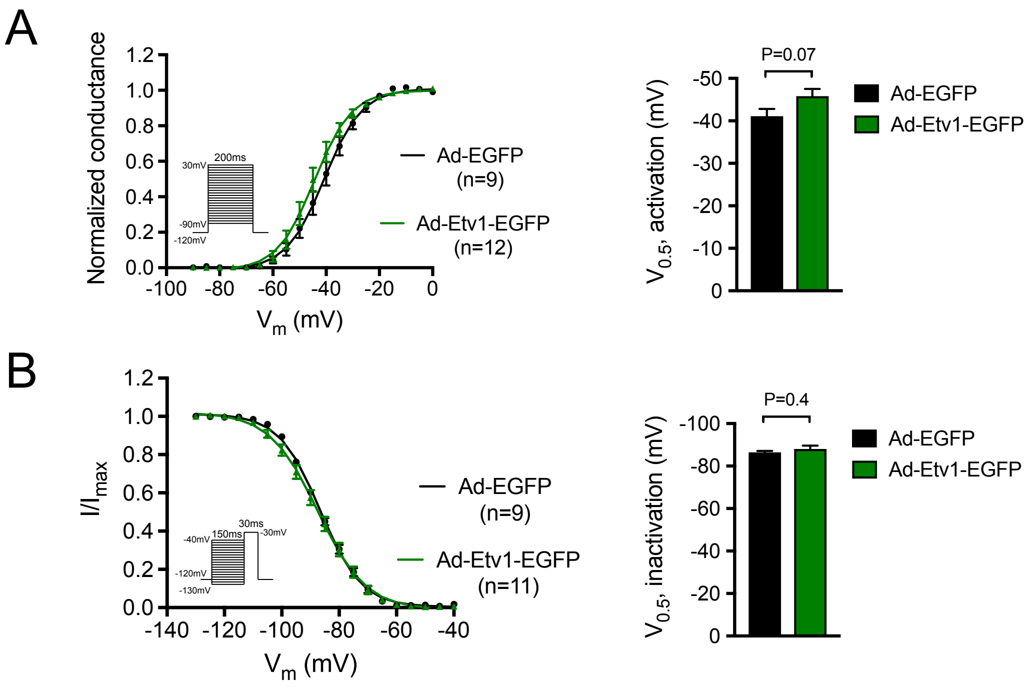

Supplemental Figure 5. Sodium channel biophysical properties in human induced pluripotent stem cells-derived cardiomyocytes transduced with ETV1. (A) Voltage dependence of steady-state activation. (B) Voltage dependence of steady-state inactivation. (A-B) Voltage at half activation ($V_{0.5}$, activation) and voltage at half inactivation ($V_{0.5}$, inactivation) were used to assess significant differences among experimental groups (right panels). Diagrams outlining patch clamp protocols are included for each endpoint. Data represent mean ± SEM. *P < 0.05, two-tailed Student's t-test.

Human ETV1

```
        10         20         30         40         50
MDGFYDQQVP YMVTNSQRGR NCNEKPTNVR KRKFINRDLA HDSEELFQDL
        60         70         80         90        100
SQLQETWLAE AQVPDNDEQF VPDYQAESLA FHGLPLKIKK EPHSPCSEIS
       110        120        130        140        150
SACSQEQPFK FSYGEKCLYN VSAYDQKPQV GMRPSNPPTP SSTPVSPLHH
       160        170        180        190        200
ASPNSTHTPK PDRAFPAHLP PSQSIPDSSY PMDHRFRRQL SEPCNSFPPL
       210        220        230        240        250
PTMPREGRPM YQRQMSEPNI PFPPQGFKQE YHDPVYEHNT MVGSAASQSF
       260        270        280        290        300
PPPLMIKQEP RDFAYDSEVP SCHSIYMRQE GFLAHPSRTE GCMFEKGPRQ
       310        320        330        340        350
FYDDTCVVPE KFDGDIKQEP GMYREGPTYQ RRGSLQLWQF LVALLDDPSN
       360        370        380        390        400
SHFIAWTGRG MEFKLIEPEE VARRWGIQKN RPAMNYDKLS RSLRYYYEKG
       410        420        430        440        450
IMQKVAGERY VYKFVCDPEA LFSMAFPDNQ RPLLKTDMER HINEEDTVPL
       460        470
SHFDESMAYM PEGGCCNPHP YNEGYVY
```

Mouse ETV1

```
        10         20         30         40         50
MDGFYDQQVP YVVTNSQRGR NCTEKPTNVR KRKFINRDLA HDSEELFQDL
        60         70         80         90        100
SQLQETWLAE AQVPDNDEQF VPDYQAESLA FHGLPLKIKK EPHSPCSELG
       110        120        130        140        150
SACSQEQPFK FSYGEKCLYN VSAYDQKPQV GMRPSNPPTP SSTPVSPLHH
       160        170        180        190        200
ASPNTAHTPK PDRAFPAHLP PSQSIPDSTY PMDHRFRRQL SEPCNSFPPL
       210        220        230        240        250
PTMPREGRPM YQRQMSEPNI PFPPQGFKQE YHDPVYEHTT MVGGAASQSF
       260        270        280        290        300
PPPLMIKQEP RDFAYDSEVP SCHSIYMRQE GFLAHPSRTE GCMFEKGPRQ
       310        320        330        340        350
FYDDTCVVPE KFDGDIKQEP GMYREGPTYQ RRGSLQLWQF LVALLDDPSN
       360        370        380        390        400
SHFIAWTGRG MEFKLIEPEE VARRWGIQKN RPAMNYDKLS RSLRYYYEKG
       410        420        430        440        450
IMQKVAGERY VYKFVCDPEA LFSMAFPDNQ RPLLKTDMER HINEEDTVPL
       460        470
SHFDESMTYM PEGGCCNPHP YNEGYVY
```

Supplemental Figure 6. Protein sequence of human ETV1 and mouse ETV1. Amino acid sequence of human and mouse ETV1 from the N-terminal to C-terminal end. Yellow highlighted and bolded sequence distinguishes the core DNA binding (ETS) region. Red highlighted amino acids indicate mismatches in mouse ETV1 compared to human ETV1.

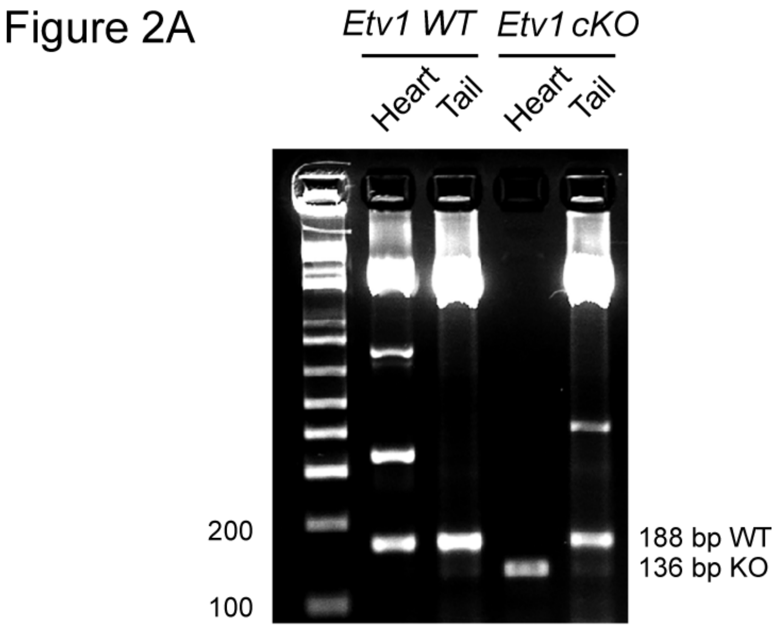

Supplemental Figure 7. Figure 2A full-length gel.

Figure 2B

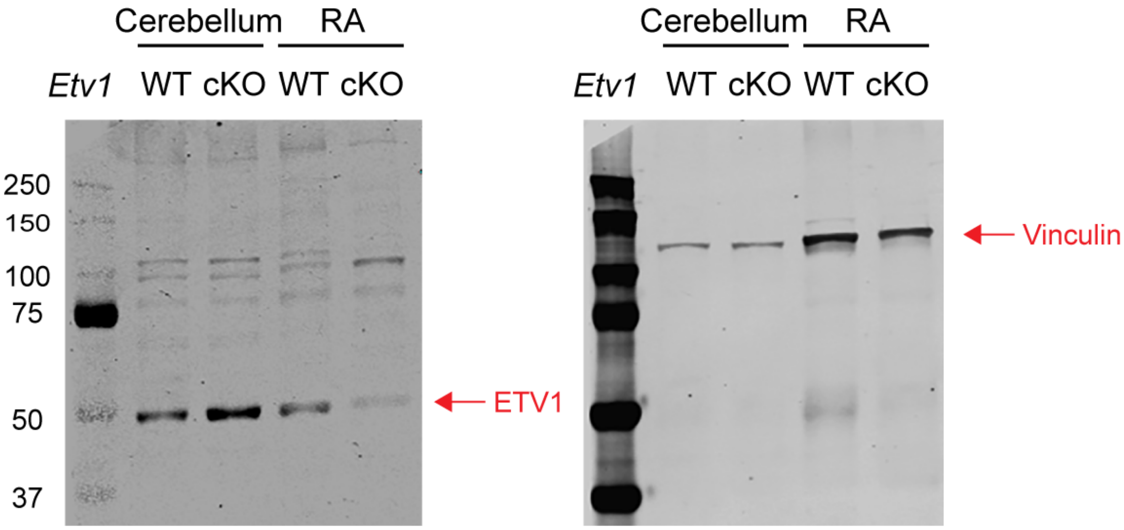

Supplemental Figure 8. Figure 2B full-length western blot.

Figure 3B

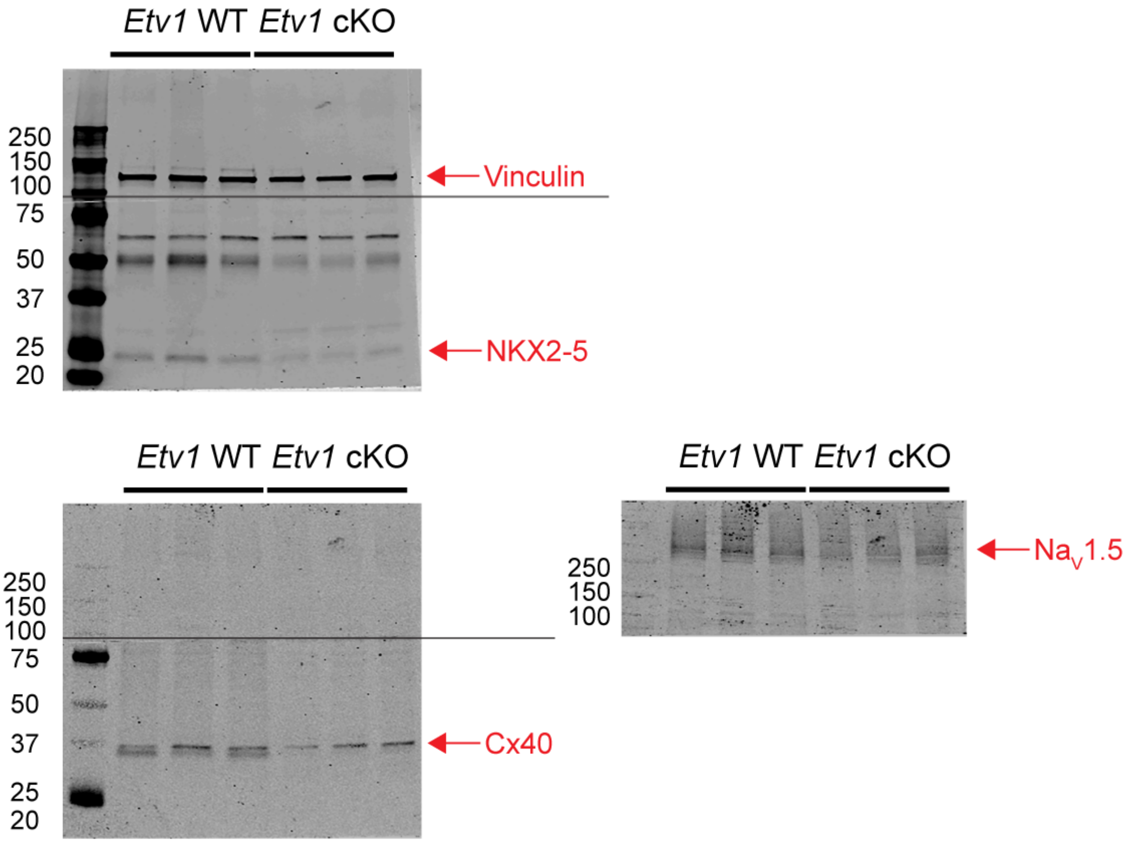

Supplemental Figure 9. Figure 3B full-length western blot.

Figure 7B

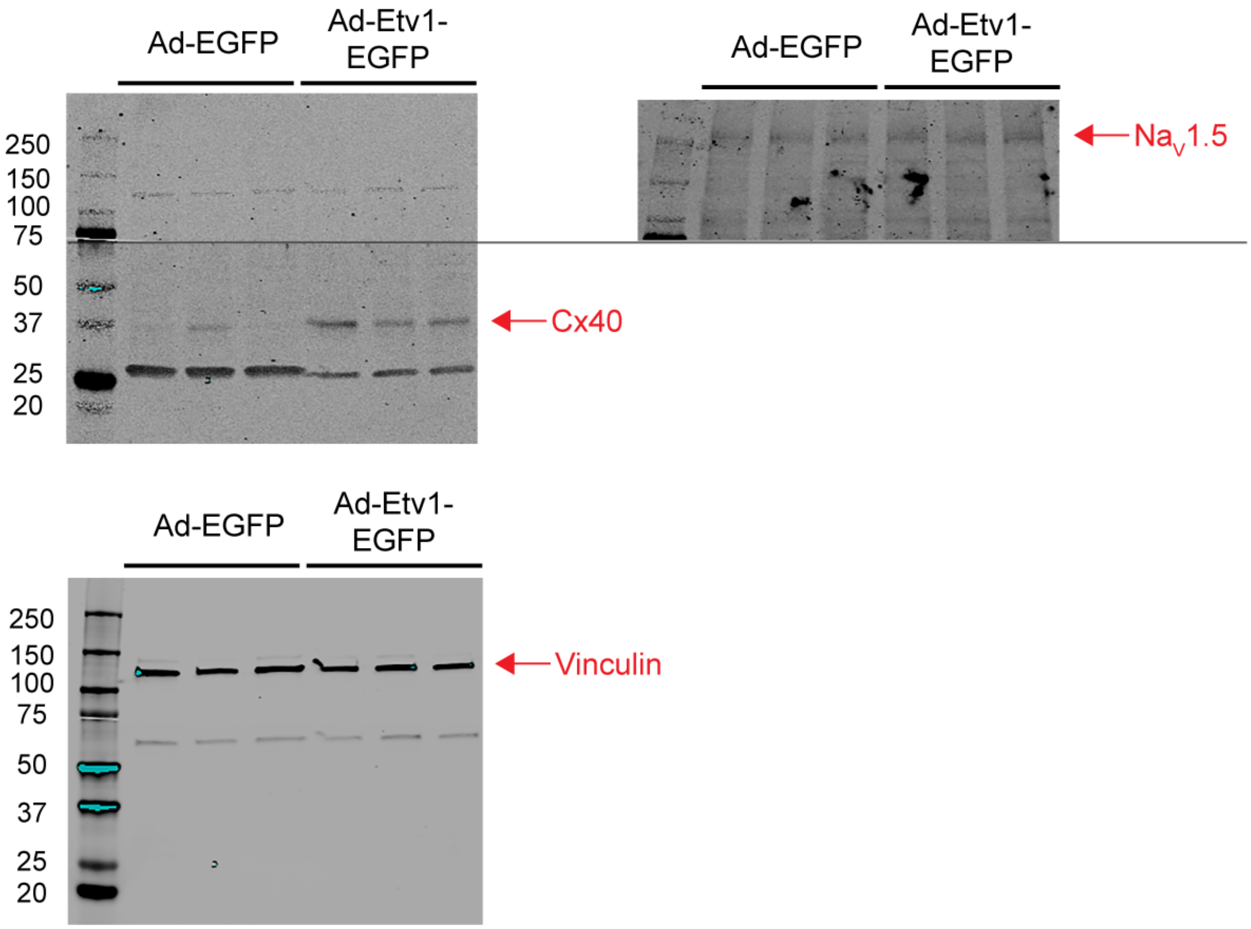

Supplemental Figure 10. Figure 7B full-length western blot.

Supplemental Tables

Supplemental Table 1: Left ventricular cardiac function and wall thickness were obtained in 10-12 week old *Etv1*$^{fl/fl}$ (WT) and *Etv1*$^{fl/fl}$; *Myh6-Cre* (cKO) mice via echocardiography. Numbers in parentheses indicate the number of mice recorded. EDV, End diastole volume; ESV, End systolic volume; SV, stroke volume; EF, ejection fraction; LV, left ventricle; AW, anterior wall; PW, posterior wall; d, diastole; s, systole. Data represent mean ± SEM.

Echocardiographic parameters in 10-12 week old *Etv1*$^{fl/fl}$ (WT) and *Etv1*$^{fl/fl}$*, Myh6-Cre* (cKO) mice

	**EDV (ul)**	**ESV (ul)**	**SV (ul)**	**EF (%)**	**LVAW,d (mm)**	**LVAW,s (mm)**	**LVPW,d (mm)**	**LVPW,s (mm)**
WT (n=5)	78.5 ± 1.4	30.6 ± 0.7	48.0 ± 0.8	61.1 ± 0.5	0.81 ± 0.01	1.25 ± 0.01	0.81 ± 0.02	1.23 ± 0.01
cKO (n=5)	82.2 ± 1.2	31.5 ± 0.3	50.7 ± 1.3	61.6 ± 0.7	0.79 ± 0.01	1.25 ± 0.02	0.83 ± 0.02	1.24 ± 0.01
P value	0.10	0.29	0.14	0.61	0.09	0.92	0.55	0.40

Supplemental Table 2: Surface electrocardiograms were obtained in 10-12 week old *Etv1*$^{fl/fl}$ (WT) and *Etv1*$^{fl/fl}$; *Myh6-Cre* (cKO) mice. Numbers in parentheses indicate the number of mice recorded. HR; Heart rate. Data represent mean ± SEM.

Cardiac conduction intervals in 10-12 week old *Etv1*$^{fl/fl}$ (WT) and *Etv1*$^{fl/fl}$*, Myh6-Cre* (cKO) mice

	**HR (bpm)**	**PR (ms)**	**P (ms)**	**QRS (ms)**	**JT (ms)**	**RsR'**
WT (n=15)	456 + 15	36.7 + 0.7	13.9 + 0.4	10.6 + 0.1	8.4 + 0.3	0%
cKO (n=15)	440 + 12	35.0 + 0.7	17.9 + 0.5	13.4 + 0.2	7.8 + 0.5	20%
P value	0.30	0.15	7.0E-08	7.0E-10	0.11	-

Supplemental Table 3: Relative levels of *Nkx2-5*, *Gja5*, and *Scn5a* in FACS purified ventricular, atrial, and Purkinje myocytes from 10-12-week-old *Etv1*$^{fl/fl}$ (WT) and *Etv1*$^{fl/fl}$; *Myh6-Cre* (cKO) hearts. Quantitative RT-PCR of transcript levels (normalized to GAPDH), presented relative to WT. Data represent mean ± SEM.

	Nkx2-5	*Gja5*	*Scn5a*
VM *Etv1* WT (n=4)	1.00 ± 0.09	-	1.00 ± 0.09
VM *Etv1* cKO (n=4)	0.95 ± 0.09	-	1.01 ± 0.09
RA *Etv1* WT (n=4)	1.00 ± 0.08	1.00 ± 0.06	1.00 ± 0.10
RA *Etv1* cKO (n=4)	0.53 ± 0.04*	0.49 ± 0.06*	0.56 ± 0.06*
PC *Etv1* WT (n=4)	1.00 ± 0.07	1.00 ± 0.08	1.00 ± 0.12
PC *Etv1* cKO (n=4)	0.53 ± 0.09*	0.55 ± 0.09*	0.61 ± 0.09*

*P<0.05 vs WT

Supplemental Table 4: Relative levels of NKX2-5, Cx40, and $Na_V1.5$ in atrial myocytes from 10-12-week-old *Etv1*$^{fl/fl}$ (WT) and *Etv1*$^{fl/fl}$; *Myh6-Cre* (cKO) hearts. Western blot densitometry quantification of protein levels (normalized to Vinculin), presented relative to WT. Data represent mean ± SEM.

	NKX2-5	Cx40	$Na_V1.5$
VM *Etv1* WT (n=5)	1.00 ± 0.19	1.00 ± 0.08	1.00 ± 0.08
VM *Etv1* cKO (n=5)	0.57 ± 0.09*	0.48 ± 0.16*	0.64 ± 0.05*

*P<0.05 vs WT

Supplemental Table 5: Sodium channel biophysical properties between ventricular, atrial, and Purkinje myocytes from 10-12-week-old *Etv1*$^{fl/fl}$ (WT) and *Etv1*$^{fl/fl}$; *Myh6-Cre* (cKO) mice.

	Etv1 WT (N=4 mice)			*Etv1* cKO (N=4 mice)		
	VM	RA	PC	VM	RA	PC
Maximum Conductance (nS/pF)	0.92 ± 0.04 (n=10)	1.14 ± 0.07 (n=13)	1.34 ± 0.06 (n=13)	0.93 ± 0.05 (n=12)	0.84 ± 0.07 (n=13)	1.02 ± 0.07 (n=10)
$V_{0.5}$, activation (mV)	-51.6 ± 1.2 (n=10)	-54.6 ± 1.4 (n=13)	-55.9 ± 0.9 (n=13)	-49.4 ± 1.2 (n=12)	-48.6 ± 1.1 (n=13)	-50.7 ± 1.5 (n=10)
$V_{0.5}$, inactivation (mV)	-83.2 ± 1.7 (n=10)	-90.1 ± 1.3 (n=11)	-83.9 ± 1.3 (n=12)	-82.2 ± 1.1 (n=13)	-84.8 ± 1.0 (n=11)	-84.3 ± 0.5 (n=12)
Tau of recovery from inactivation (ms)	2.1 ± 0.1 (n=10)	1.8 ± 0.1 (n=10)	1.8 ± 0.1 (n=11)	2.1 ± 0.1 (n=9)	2.2 ± 0.1 (n=11)	2.2 ± 0.1 (n=10)

*Statistics outlined in Supplemental tables 6-9

Supplemental Table 6: One-way ANOVA of sodium channel maximum conductance (nS/pF).

One-way ANOVA Max. Conductance (nS/pF)	Mean Diff.	95.00% CI of diff.	Summary	P Value
Etv1 WT VM vs. *Etv1* WT RA	-0.2188	-0.4013 to -0.03636	*	0.0195
Etv1 WT VM vs. *Etv1* WT PC	-0.424	-0.6065 to -0.2415	****	<0.0001
Etv1 WT RA vs. *Etv1* WT PC	-0.2052	-0.3753 to -0.03505	*	0.0188
Etv1 cKO VM vs. *Etv1* cKO RA	0.08375	-0.0869 to 0.2544	ns	0.3307
Etv1 cKO VM vs. *Etv1* cKO PC	-0.08955	-0.2753 to 0.09618	ns	0.3392
Etv1 cKO RA vs. *Etv1* cKO PC	-0.1733	-0.3529 to 0.006301	ns	0.0583
Etv1 WT VM vs. *Etv1* cKO VM	-0.01022	-0.196 to 0.1755	ns	0.9128
Etv1 WT RA vs. *Etv1* cKO RA	0.2923	0.1253 to 0.4594	***	0.0009
Etv1 WT PC vs. *Etv1* cKO PC	0.3242	0.1418 to 0.5067	***	0.0007

Supplemental Table 7: One-way ANOVA of sodium channel $V_{0.5}$, activation (mV).

One-way ANOVA $V_{0.5}$, activation (mV)	Mean Diff.	95.00% CI of diff.	Summary	P Value
Etv1 WT VM vs. *Etv1* WT RA	3.004	-0.6139 to 6.621	ns	0.1021
Etv1 WT VM vs. *Etv1* WT PC	4.336	0.7819 to 7.889	*	0.0176
Etv1 WT RA vs. *Etv1* WT PC	1.332	-2.05 to 4.714	ns	0.4343
Etv1 cKO VM vs. *Etv1* cKO RA	-0.8752	-4.257 to 2.507	ns	0.607
Etv1 cKO VM vs. *Etv1* cKO PC	1.294	-2.324 to 4.911	ns	0.4775
Etv1 cKO RA vs. *Etv1* cKO PC	2.169	-1.385 to 5.723	ns	0.2272
Etv1 WT VM vs. *Etv1* cKO VM	-2.112	-5.729 to 1.506	ns	0.2479
Etv1 WT RA vs. *Etv1* cKO RA	-5.991	-9.373 to -2.608	***	0.0008
Etv1 WT PC vs. *Etv1* cKO PC	-5.154	-8.707 to -1.6	**	0.0052

Supplemental Table 8: One-way ANOVA of sodium channel $V_{0.5}$, inactivation (mV).

One-way ANOVA $V_{0.5}$, inactivation (mV)	Mean Diff.	95.00% CI of diff.	Summary	P Value
Etv1 WT VM vs. *Etv1* WT RA	6.876	3.425 to 10.33	***	0.0002
Etv1 WT VM vs. *Etv1* WT PC	0.7015	-2.681 to 4.084	ns	0.6799
Etv1 WT RA vs. *Etv1* WT PC	-6.175	-9.472 to -2.877	***	0.0004
Etv1 cKO VM vs. *Etv1* cKO RA	2.57	-0.6666 to 5.806	ns	0.1176
Etv1 cKO VM vs. *Etv1* cKO PC	2.104	-1.058 to 5.267	ns	0.1884
Etv1 cKO RA vs. *Etv1* cKO PC	-0.4652	-3.763 to 2.832	ns	0.7789
Etv1 WT VM vs. *Etv1* cKO VM	-1.007	-4.33 to 2.316	ns	0.547
Etv1 WT RA vs. *Etv1* cKO RA	-5.314	-8.682 to -1.945	**	0.0025
Etv1 WT PC vs. *Etv1* cKO PC	0.396	-2.829 to 3.621	ns	0.807

Supplemental Table 9: One-way ANOVA of sodium channel Tau of recovery from inactivation (ms)

One-way ANOVA<br>$V_{0.5}$, inactivation (mV)	Mean Diff.	95.00% CI of diff.	Summary	P Value
Etv1 WT VM vs. *Etv1* WT RA	0.3133	0.07193 to 0.5547	*	0.0119
Etv1 WT VM vs. *Etv1* WT PC	0.2711	0.03551 to 0.5066	*	0.0249
Etv1 WT RA vs. *Etv1* WT PC	-0.04224	-0.2836 to 0.1991	ns	0.7273
Etv1 cKO VM vs. *Etv1* cKO RA	-0.09034	-0.3386 to 0.1579	ns	0.4691
Etv1 cKO VM vs. *Etv1* cKO PC	-0.1259	-0.3797 to 0.1279	ns	0.3245
Etv1 cKO RA vs. *Etv1* cKO PC	-0.03559	-0.277 to 0.2058	ns	0.7688
Etv1 WT VM vs. *Etv1* cKO VM	0.01045	-0.2378 to 0.2587	ns	0.9331
Etv1 WT RA vs. *Etv1* cKO RA	-0.3932	-0.6345 to -0.1518	**	0.0019
Etv1 WT PC vs. *Etv1* cKO PC	-0.3865	-0.6279 to -0.1452	**	0.0022

Supplemental Table 10: Gene expression comparison between FACS purified postnatal day 21 (P21) Purkinje and myocytes vs Ad-ETV1-EGFP or Ad-EGFP transduced postnatal day 1 NRVMs. Quantitative RT-PCR (qRT-PCR) of transcript levels (normalized to *Gapdh*), presented relative to myocytes or Ad-EGFP respectively. Data represent mean ± SEM.

Gene Expression changes in WT mouse Purkinje vs ventricular myocytes

Gene	P21 WT Purkinje vs Myocyte Log_2 fold change by RNA-seq	Padj (RNA-seq)	P21 WT Purkinje vs Myocyte Log_2 fold change by qRT-PCR	*P* (qRT-PCR)
Etv1	4.23	3.6E-47	4.66 ± 0.18	4.6E-06
Tbx5	1.27	3.8E-09	1.50 ± 0.19	0.00014
Nkx2-5	0.35	0.37	0.58 ± 0.20	0.031
Irx3	0.71	0.16	1.08 ± 0.34	0.047
Irx5	1.26	0.0068	1.93 ± 0.32	0.0038
Ache	4.59	6.2E-19	7.23 ± 0.47	0.0055
Scn5a	0.48	0.012	0.57 ± 0.16	0.0098
Gja5	3.08	1.2E-07	7.38 ± 0.24	8.7E-05
Hcn4	2.83	3.4E-18	3.45 ± 0.22	0.00012
Tbx18	-0.29	0.78	-0.51 ± 0.55	0.20
Tbx20	-0.74	3.7E-05	-0.87 ± 0.15	3.3E-05
Irx4	-0.42	0.31	-0.59 ± 0.32	0.034

Gene Expression changes in Ad-Etv1-EGFP vs Ad-EGFP transduced P1 NRVMs

Gene	P1 NRVM Ad-Etv1 vs Ad-EGFP Log_2 fold change by RNA-seq	Padj (RNA-seq)	P1 NRVM Ad-Etv1 vs Ad-EGFP Log_2 fold change by qRT-PCR	*P* (qRT-PCR)
Etv1	5.33	< 1.00E-300	5.61 ± 0.13	4.8E-05
Tbx5	0.83	1.3E-13	0.94 ± 0.14	0.0057
Nkx2-5	0.65	5.4E-27	1.01 ± 0.24	0.019
Irx3	0.81	2.1E-21	0.62 ± 0.10	0.014
Irx5	1.25	4.7E-32	1.51 ± 0.22	0.0042
Ache	2.58	6.6E-74	2.85 ± 0.18	1.1E-04
Scn5a	1.62	5.0E-140	2.19 ± 0.12	0.017
Gja5	1.40	1.8E-87	3.28 ± 0.13	0.027
Hcn4	0.41	1.4E-04	0.52 ± 0.12	0.015
Tbx18	-0.84	1.1E-17	-0.67 ± 0.19	0.027
Tbx20	-0.95	3.7E-69	-1.04 ± 0.12	0.0019
Irx4	-0.69	7.0E-12	-0.83 ± 0.24	0.029

Supplemental Table 11: Relative levels of *Etv1*, *NKX2-5*, *GJA5*, *SCN5A, and MYL2* in Ad-EGFP or Ad-Etv1-EGFP transduced human induced pluripotent stem cell-derived cardiomyocytes (hiPSC-CM). Quantitative RT-PCR of transcript levels (normalized to *GAPDH*), presented relative to WT. Data represent mean ± SEM.

Gene Expression changes in Ad-Etv1-EGFP vs Ad-EGFP transduced hiPSC-CM

**Gene**	**hiPSC-CM Ad-Etv1-EGFP vs Ad-EGFP Log_2 fold change by qRT-PCR**	***P* (qRT-PCR)**
Etv1	6.13 ± 0.06	2.2E-07
NKX2-5	1.50 ± 0.26	9.5E-04
GJA5	2.37 ± 0.20	4.6E-03
SCN5A	1.73 ± 0.16	9.9E-04
MYL2	-0.26 ± 0.12	0.26

Supplemental Table 12: Sodium channel biophysical properties between Ad-EGFP or Ad-Etv1-EGFP transduced human induced pluripotent stem cell-derived cardiomyocytes. Data represent mean ± SEM.

	Ad-EGFP	Ad-Etv1-EGFP	P Value
Maximum Conductance (nS/pF)	1.26 ± 0.14 (n=9)	2.52 ± 0.33 (n=12)	0.007
$V_{0.5}$, activation (mV)	-41.1 ± 1.2 (n=9)	-45.8 ± 1.6 (n=12)	0.07
$V_{0.5}$, inactivation (mV)	-86.4 ± 0.7 (n=9)	-88.0 ± 1.5 (n=11)	0.4

Supplemental Table 13: Oligonucleotides for quantitative real time PCR analysis
OriGene Technologies (MP205604): product length = 153
Mouse *Gapdh* F 5'-CATCACTGCCACCCAGAAGACTG–3'
Mouse *Gapdh* R 5'-ATGCCAGTGAGCTTCCCGTTCAG–3'

product length = 153
Rat *Gapdh* F 5'-CATCACTGCCACTCAGAAGACTG–3'
Rat *Gapdh* R 5'-ATGCCAGTGAGCTTCCCGTTCAG–3'

OriGene Technologies (HP205798): product length = 153
Human *GAPDH* F 5'-CATCACTGCCACTCAGAAGACTG–3'
Human *GAPDH* R 5'-ATGCCAGTGAGCTTCCCGTTCAG–3'

OriGene Technologies (MP204358): product length = 127
Mouse *Etv1* F 5'-TCCTGGCTCATCCAAGCAGAAC-3'
Mouse *Etv1* F 5'-CGGTACATTCCAGGCTCTTGCT-3'

product length = 127
Rat *Etv1* F 5'-TCCTGGCTCATCCAAGCAGAAC-3'
Rat *Etv1* F 5'-CGGTACATTCCTGGCTCTTGCT-3'

product length = 110
Mouse *Tbx5* F 5'-GGCATGGAAGGAATCAAGGT-3'
Mouse *Tbx5* R 5'-CTAGGAAACATTCTCCTCCCTGC-3'

product length = 110
Rat *Tbx5* F 5'-GGCATGGAAGGAATCAAGGT-3'
Rat *Tbx5* R 5'-CTAGGAAACATCTGCCTCCCTGC-3'

product length = 227
Mouse *Nkx2-5* F 5-ACCTTTCTCCGATCCATCCCACTT-3
Mouse *Nkx2-5* R 5-GCGTTAGCGCACTCACTTTAATGG-3

product length = 140
Rat *Nkx2-5* F 5-GGATTTCACACCCACACTTGC-3
Rat *Nkx2-5* R 5-TCCGGGTCCTGATATGGAATC-3

OriGene Technologies (HP207706): product length = 146
Human *NKX2-5* F 5'-AAGTGTGCGTCTGCCTTTCCCG–3'
Human *NKX2-5* R 5'-TTGTCCGCCTCTGTCTTCTCCA–3'

OriGene Technologies (MP206854):product length = 124
Mouse *Irx3* F 5-AGCCGCAGGTCATCCCGCTG-3
Mouse *Irx3* R 5-CGGCTGGAAAGCTGTCTTGAGT-3

product length = 124
Rat *Irx3* F 5-AGCCGCAGGCCACCCCGCTG-3
Rat *Irx3* R 5-CGGCTGGAAAGCTGTCTTAAGT-3

OriGene Technologies (MP206856): product length = 139

Mouse *Irx5* F 5-TACAACTCGCACCTCCAGTACG-3
Mouse *Irx5* R 5-AGCCCAGAGGTGCTGCATAAGG-3

product length = 139
Rat *Irx5* F 5-TACAACTCGCACCTCCAGTACG-3
Rat *Irx5* R 5-AGCCCAGAGGTGCGGCGTAAGG-3

OriGene Technologies (MP200188): product length = 135
Mouse *Ache* F 5-TTCCTTCGTGCCTGTGGTAGAC-3
Mouse *Ache* R 5-CCGTAAACCAGAAAGTAGGAGCC-3

product length = 135
Rat *Ache* F 5-TTCCTTCGTGCCTGTGGTGGAC-3
Rat *Ache* R 5-CCGTAAACCAGAAAGTAGGAGCC-3

product length = 101
Mouse/Rat *Scn5a* F 5'-GAAGAAGCTGGGCTCCAAGA-3'
Mouse/Rat *Scn5a* R 5'-CATCGAAGGCCTGCTTGGTC-3'

OriGene Technologies (HP234325): product length = 145
Human *SCN5A* F 5'-CAAGACCTGCTACCACATCGTG–3'
Human *SCN5A* R 5'-GTCGGCATACTCAAGCAGAACC–3'

OriGene Technologies (MP205237): product length = 128
Mouse *Gja5* F 5'-GTGCCAAACCAGGAGCAGATTC–3'
Mouse *Gja5* R 5'-CGCCGTTTGTCACTATGGTAGC-3'

product length = 128
Rat *Gja5* F 5'-GTGCCAAACCAGGAGCAGATCC–3'
Rat *Gja5* R 5'-CGGCGCTTGTCACTGTGGTAGC-3'

OriGene Technologies (HP208435): product length = 149
Human *GJA5* F 5'-TAGGCAAGGTCTGGCTCACTGT–3'
Human *GJA5* R 5'-GAAAGCCTGGTCGTAGCAGACA-3'

product length = 84
Mouse *Hcn4* F 5-TGGCTGATGGCTCCTATTTT-3
Mouse *Hcn4* R 5-GGCAATAAGTATCCGCTCTGA-3

product length = 145
Rat *Hcn4* F 5-TGGCTGATGGCTCCTATTTT-3
Rat *Hcn4* R 5-GGCAATAAGTATCCGCTCTGA-3

OriGene Technologies (MP216696): product length = 123
Mouse *Tbx18* F 5-GGTGGAGTCATACGCATTCTGG-3
Mouse *Tbx18* R 5-ACAGCATTCCCAGTGCCTTGGA-3

product length = 123
Rat *Tbx18* F 5-GGTGGAGTCATATGCATTCTGG-3
Rat *Tbx18* R 5-ACGCCATTCCCAGTACCTGGGA-3

OriGene Technologies (MP216688): product length = 138
Mouse/Rat *Tbx20* F 5-AGCAGTCACAGCCTACCAGAAC-3
Mouse/Rat *Tbx20* R 5-GAATGCTTCTGGATCAGGCTCTC-3

OriGene Technologies (MP206855): product length = 123
Mouse *Irx4* F 5-GTGACCTATGGCTCTGAGGCAT-3
Mouse *Irx4* R 5-CTCATACGGGTAGTAGGCTGCA-3

product length = 123
Rat *Irx4* F 5-GTGACCTATGGTTCTGAGGCAT-3
Rat *Irx4* R 5-CTCATAAGGGTAGTAGGCTGCA-3

OriGene Technologies (HP200412): product length = 113
Human *MYL2* F 5-CGGAGAGGTTTTCCAAGGAGGA-3
Human *MYL2* R 5-CTCTTCTCCGTGGGTGATGATG-3
